# Viruses in Nondisinfected Drinking Water from Municipal Wells and Community Incidence of Acute Gastrointestinal Illness

**DOI:** 10.1289/ehp.1104499

**Published:** 2012-06-01

**Authors:** Mark A. Borchardt, Susan K. Spencer, Burney A. Kieke, Elisabetta Lambertini, Frank J. Loge

**Affiliations:** 1 Marshfield Clinic Research Foundation, Marshfield, Wisconsin, USA; 2 Department of Civil and Environmental Engineering, University of California, Davis, Davis, California, USA

**Keywords:** acute gastrointestinal illness, drinking water, groundwater, public health, public water system, quantitative microbial risk assessment, quantitative polymerase chain reaction, viruses

## Abstract

Background: Groundwater supplies for drinking water are frequently contaminated with low levels of human enteric virus genomes, yet evidence for waterborne disease transmission is lacking.

Objectives: We related quantitative polymerase chain reaction (qPCR)–measured enteric viruses in the tap water of 14 Wisconsin communities supplied by nondisinfected groundwater to acute gastrointestinal illness (AGI) incidence.

Methods: AGI incidence was estimated from health diaries completed weekly by households within each study community during four 12-week periods. Water samples were collected monthly from five to eight households per community. Viruses were measured by qPCR, and infectivity assessed by cell culture. AGI incidence was related to virus measures using Poisson regression with random effects.

Results: Communities and time periods with the highest virus measures had correspondingly high AGI incidence. This association was particularly strong for norovirus genogroup I (NoV-GI) and between adult AGI and enteroviruses when echovirus serotypes predominated. At mean concentrations of 1 and 0.8 genomic copies/L of NoV-GI and enteroviruses, respectively, the AGI incidence rate ratios (i.e., relative risk) increased by 30%. Adenoviruses were common, but tap-water concentrations were low and not positively associated with AGI. The estimated fraction of AGI attributable to tap-water–borne viruses was between 6% and 22%, depending on the virus exposure–AGI incidence model selected, and could have been as high as 63% among children < 5 years of age during the period when NoV-GI was abundant in drinking water.

Conclusions: The majority of groundwater-source public water systems in the United States produce water without disinfection, and our findings suggest that populations served by such systems may be exposed to waterborne viruses and consequent health risks.

More than 70 years ago, Kling (1939) linked poliomyelitis with drinking contaminated well water. Since then numerous environmental studies have detected nearly every known human enteric virus in groundwater supplies around the world (e.g., Abbaszadegan et al. 2003; Borchardt et al. 2007; Fout et al. 2003; Lee et al. 2011; Locas et al. 2007; Powell et al. 2003), yet the public health significance is still not clearly understood. Virus-contaminated groundwater is noted for causing acute gastrointestinal illness (AGI) outbreaks. Of the 36 outbreaks associated with drinking water reported in the United States in 2007–2008, 22 (61%) were from groundwater systems, including 5 outbreaks (23%) caused by viruses (Brunkard et al. 2011). However, whether virus-contaminated groundwater is responsible for sporadic and endemic AGI is unknown.

Because of their small size (i.e., 25–90 nm), low attachment to particles at typical soil pH (Gupta et al. 2009), and large numbers shed in the stool of infected individuals, viruses have great potential to travel downward through the soil profile, reach groundwater, and move with groundwater flow to drinking water wells. Viruses capable of causing AGI are host-specific, obligate enteric pathogens that are incapable of replicating in the environment. Consequently, pathogenic viruses contaminating a well must originate from nearby human fecal sources such as septic systems, landfills, polluted infiltrating surface water, or leaking sanitary sewers. The same viruses present in human wastewater are commonly detected in groundwater: adenovirus, enterovirus, hepatitis A virus, norovirus, and rotavirus. Infections can result in a variety of acute illnesses (e.g., AGI, fever, conjunctivitis, aseptic meningitis, hand-foot-and-mouth disease) that may be mild to severe to fatal (Knipe and Howley 2007). Chronic sequelae also occur and may involve circulatory, neurologic, or hepatic systems.

In 2006, the U.S. Environmental Protection Agency (EPA) promulgated the Groundwater Rule to mitigate the population’s exposure to groundwater-borne pathogens (U.S. EPA 2006a). There are 147,330 public water systems supplying groundwater to more than 100 million people in the United States. The majority of these public water systems (i.e., 95,631 public water systems serving 20 million people) produce water without disinfection. In addition, another 56.8 million people drink treated groundwater that does not meet the goal of reducing viruses by 99.99% (U.S. EPA 2006b). Disinfection is not an automatic Groundwater Rule requirement. The U.S. EPA estimated that 27% of public water system wells will be virus contaminated at some point in time (U.S. EPA 2006c), suggesting that a segment of the U.S. population may still have a significant exposure to groundwater-borne viruses.

Our main objective was to quantify viruses by quantitative polymerase chain reaction (qPCR) in the tap water of 14 communities that use nondisinfected groundwater as their drinking water source and to relate virus exposure levels to the community-level incidence of AGI. A secondary objective was to use quantitative microbial risk assessment (QMRA) to estimate the fraction of AGI attributable to tap-water viruses in the communities’ drinking water.

## Materials and Methods

*Study communities*. The communities (population range 1,363–8,300) were located in Wisconsin (USA), and were among 124 communities in the state that used nondisinfected groundwater at the time the study began. We solicited communities with populations > 1,000 and with ≤ four wells, and we enrolled the first 14 communities whose governing boards (e.g., city councils) approved participation. The communities’ municipally owned drinking water utilities met all requirements under the Safe Drinking Water Act Amendments (1996). Drinking water was supplied by wells drilled 23–169 m in various hydrogeological settings, primarily sandstone aquifers. All the wells were classified as free of surface water influence and therefore not subject to any treatment requirements. There were no centralized drinking-water treatment plants. Disinfection was absent except for occasional short-term chlorination events during routine maintenance or if there was an acute violation of the federally regulated maximum contaminant level for coliform bacteria or *Escherichia coli.* The communities’ water systems have been described further by Lambertini et al. (2011).

*Study design*. The work described herein is part of the Wisconsin Water And Health Trial for Enteric Risk (WAHTER) study, designed to address several questions related to drinking water–borne disease. The overarching study design was a randomized community-intervention trial with crossover. Intervention was by low-pressure, ultraviolet light (UV) disinfection reactors (WEDECO, Charlotte, NC) that were installed on all operating municipal wells to treat the water at a dose of 50 mJ/cm^2^. Eight communities had UV reactors installed the first study year (2006), while the six remaining communities continued to use nondisinfected water. Crossover was implemented in the winter of 2006–2007 by transferring the UV reactors so that in the second study year (2007) the six communities had UV-disinfected drinking water while the eight communities resumed drinking nondisinfected water. The intervention was designed to allow estimation of the proportion of AGI resulting from drinking pathogen-contaminated groundwater. In the present study, we focused on virus exposure from tap water. We included all tap-water samples in the present analysis regardless of whether samples were collected during intervention or control periods. Approximately one-half of the tap-water samples per community were collected during UV disinfection. UV does not possess any residual disinfecting activity, and it only inactivates viruses pumped from the well. Therefore, without chlorine in the nondisinfecting systems, it was still possible for viruses to enter the distribution system piping directly and be present in tap water during UV intervention periods (Lambertini et al. 2011).

*Epidemiological data collection.* We prospectively measured AGI in 621 households in the 14 communities during four 12-week surveillance periods: *a*) April–June 2006, *b*) September–November 2006, *c*) March–May 2007, and *d*) September–November 2007. The surveillance periods were selected to overlap with Wisconsin spring and autumn peaks in rotavirus and enterovirus infections, respectively (Nelson et al. 1979; Török et al. 1997). Summer was skipped because prestudy focus groups indicated this was when participant dropout would be greatest; winter surveillance was skipped because this season, when construction slows, was the most affordable time for installing the UV disinfection units. Eligible households had to be connected to the municipal water system and have at least one child between 6 months and 12 years of age. Children > 12 years of age were not enrolled. We identified households from water utility billing addresses. Participation by one adult household member was also requested. People with any chronic gastrointestinal illness (e.g., Crohn’s disease) and children attending for ≥ 20 hr/week a school or daycare that was not serviced by the municipal water system were excluded. See Supplemental Material, Figure S1 (http://dx.doi.org/10.1289/ehp.1104499) for a flow chart of the number of households and participants during the recruitment process. An adult completed a daily checklist recording AGI symptoms (e.g., diarrhea, vomiting) for every participating household member. Checklists were mailed to the study team weekly; if necessary, adult recorders were reminded by telephone to return checklists. Checklists that were received ≥ 21 days from the end of the weekly reporting period were excluded from analysis [2,821/64,265 (4.4%) were excluded]. Illness episodes were considered distinct when separated by ≥ 6 symptom-free days. AGI was defined as having three or more episodes of loose watery stools or one vomiting episode in a 24-hr period. We considered time spent away from home in our calculation of person-time at-risk. Person-time, the time considered at-risk for an AGI episode from drinking water exposure, was estimated by having participants report on the checklist the nights that they slept away from home. Days immediately preceded by 3 consecutive not-at-home days were classified as not-at-risk because AGI episodes beginning on such days were likely due to exposures outside the community. The research protocol was approved by Marshfield Clinic (Marshfield, WI) institutional review board; informed consent was provided by all participants.

*Virus sampling*. We sampled monthly for viruses in tap water from five to eight households per community during the 12-week AGI surveillance periods (i.e., three sample times per period, resulting in 17–24 samples per community per period). Our goal was to characterize the virus exposure level in a community’s drinking water, and the homes of study participants made convenient sampling locations. Households were selected using utility-provided maps of drinking water main pipes, striving to create a sample set spatially representative of the entire distribution system. We also sampled well water immediately after UV disinfection (before it entered the distribution system) on the same days that household samples were obtained in order to quantify viruses that were inactivated by UV treatment (i.e., incapable of causing AGI) but still PCR amplifiable. Viruses were concentrated by trained staff using glass wool filters attached to taps (Lambertini et al. 2008). Taps were flushed several minutes before sampling. Sample flow rate was 4 L/min and the mean sample volume was 863 L (*n* = 1,204). Glass wool filters were transported on ice to the laboratory within 48 hr of collection.

*Virus analyses*. Viruses were eluted from the filters with beef extract and additionally concentrated by polyethylene glycol flocculation using previously described methods (Lambertini et al. 2008). The 2-mL final concentrated sample volume was stored at –80°C. After nucleic acid extraction, RNA viruses were reverse-transcribed and qPCR performed with the LightCycler 480 instrument (Roche Diagnostics, Mannheim, Germany) using the LightCycler 480 Probes Master kit.

Inhibition was measured on every sample and, if necessary, mitigated by dilution with nuclease-free water. Of the 1,204 tap-water samples analyzed, only 94 required dilution.

We performed qPCR analysis twice on each sample. If both duplicates were negative, the result was reported as zero. If only one was positive, this positive virus concentration was reported. If both duplicates were positive, the average was reported. [See Supplemental Material, pp. 5–12 (http://dx.doi.org/10.1289/ehp.1104499) for details regarding sampling and qPCR quality controls, primers and probes (Supplemental Material, Table S1), inhibition measurement, standard curve preparation, quality assurance parameters (Supplemental Material, Table S2), and calculations for virus concentrations.]

All adenoviruses and enteroviruses in qPCR-positive samples were serotyped by nucleotide sequencing [see Supplemental Material, p. 13–14 (http://dx.doi.org/10.1289/ehp.1104499)].

Additionally, all adenovirus and enterovirus qPCR–positive samples were further evaluated for virus infectivity by cell culture [see Supplemental Material, p. 13 (http://dx.doi.org/10.1289/ehp.1104499)]. We used three cell lines for enteroviruses (BGM, RD, and CaCo-2 cell lines) and two for adenoviruses (Graham 293 and A549 cell lines). All cultures were passaged three times without producing cytopathic effect. At the conclusions of the second passage (4 weeks) and third passage (6 weeks), all cell lysates were analyzed by qPCR for enterovirus and adenovirus using the methods described above. When the viral gene target quantity measured in cell culture was > 10 times more than the quantity present in the initial final concentrated sample volume inoculum, the virus was considered to have multiplied in cell culture without producing cytopathic effect, and the sample was designated positive for infectious virus by integrated cell culture–qPCR (ICC-qPCR).

*Statistical methods*. We used Poisson regression with offsets to model the natural logarithm of AGI episodes per person-day of at-risk follow-up as a function of virus exposure. AGI episodes and person-days at-risk were summed within community and surveillance period resulting in 56 (14 × 4) incidence estimates for each of four age groups: all ages, adults (19–74 years of age), children 6–12 years of age, and children ≤ 5 years of age. Virus measurements were aggregated at the same level as AGI incidence, by community and 12-week surveillance period. Surveillance period, with its clearly defined start and end dates, was selected *a priori* as the least arbitrary and subjective time period for aggregating the data. Moreover, from our previous experience with sampling groundwater, we concluded that multiple water samples over time would be necessary to accurately characterize virus exposure. Aggregating by 12-week surveillance period was a sensible means of addressing this issue. The approximately 3-month gap between surveillance periods, and hence between the exposure measurements used to create the four data points for each community in the analyses, was also desirable.

Viruses were characterized by three exposure measures: arithmetic mean concentration, maximum concentration, and the proportion of virus-positive samples. These measures were calculated for four virus categories: all viruses (i.e., any virus-positive sample), norovirus genogroup I, adenovirus, and enterovirus. For the all-viruses category, virus concentrations in samples positive for more than one virus type were calculated as the sum of the numbers of each virus divided by the sample volume. Samples with no detected viruses were assigned a zero value and included in the analysis.

Model offsets consisted of the natural logarithm of the amount of at-risk follow-up time within each community and surveillance period. The term “at-risk” means eligible for a new AGI episode that could be associated with drinking water exposure within the community. AGI incidence per person-day was converted to incidence per person-year for clarity of presentation. All models included an overdispersion component in the variance function and a fixed effect for virus concentration level. Initially, this fixed effect was characterized as a restricted quadratic spline with knots at the quartiles of the positive virus concentration values (Greenland 1995). Plots derived from the models with splines were examined to assess whether a more parsimonious representation of the virus exposure effect was reasonable. After examining the data, it was deemed appropriate to use a linear (in the log of the AGI incidence) representation when evaluating overall trends. These models containing only the fixed virus concentration effect (in linear or spline form) are referred to as unadjusted. Adjusted models additionally included normally distributed random intercepts for community and surveillance period. The random intercepts for community accounted for underlying differences in AGI incidence across communities and the correlation within a given community across surveillance periods. Similarly, the random intercepts for surveillance period accounted for underlying differences in AGI incidence across surveillance periods and the correlation within a given surveillance period across communities. Unadjusted and adjusted models were fit for each virus exposure measure and the four age groups. [See Supplemental Material, p. 14–15 (http://dx.doi.org/10.1289/ehp.1104499), for additional information on model interpretation.]

We derived AGI incidence rate ratios (IRRs) from the models with splines and identified corresponding threshold points. The AGI IRR is an estimate of relative risk defined as the estimated AGI person-time incidence rate at a given level of virus exposure divided by the estimated AGI person-time incidence rate for nondetects. The threshold point is the lowest virus exposure level at which the lower confidence limit for the IRR exceeds the null value of 1.0. Ninety-five percent point-wise confidence intervals for estimated AGI incidence rates and IRRs based on the fixed effect for virus exposure were computed. Analyses were carried out with SAS (version 9.2; SAS Institute Inc., Cary, NC).

*QMRA*. We conducted Monte Carlo simulation where for every iteration a 12-week arithmetic mean concentration was calculated from single-sample virus concentration values randomly selected from the data set. The mean concentration was input into a selected Poisson regression virus exposure–AGI incidence model. The exposure–response relationship included a normally distributed error term (mean = 0), which was randomly drawn at the same time. The output represented total AGI incidence (I_T_), that is, illnesses from all sources including tap-water–borne viruses (i.e., exposed). Next, a zero concentration value was input into the virus exposure–AGI incidence model along with a random error term to obtain the baseline AGI incidence (I_B_) when tap-water–borne viruses were absent (i.e., unexposed). This value was subtracted from the total incidence, yielding an estimate of the AGI incidence rate difference (Δ) of when viruses were absent compared with when viruses were present in nondisinfected tap water. Iterations were repeated 2 × 10^5^ times. The mean or median of the frequency distribution of the incidence rate difference was divided by the mean or median of the total incidence to yield the fraction of AGI attributable to viruses in nondisinfected tap water [(I_T_ – I_B_) ÷ I_T_] (Greenland et al. 2008). See Supplemental Material, p. 15–17 (http://dx.doi.org/10.1289/ehp.1104499), for the QMRA step-by-step protocol. All model coefficients with corresponding variance/covariance estimates are reported in Supplemental Material, Table S3. The simulation was carried out in MATLAB® R2011a (MathWorks, Natick, MA).

## Results

**Figure 1:**
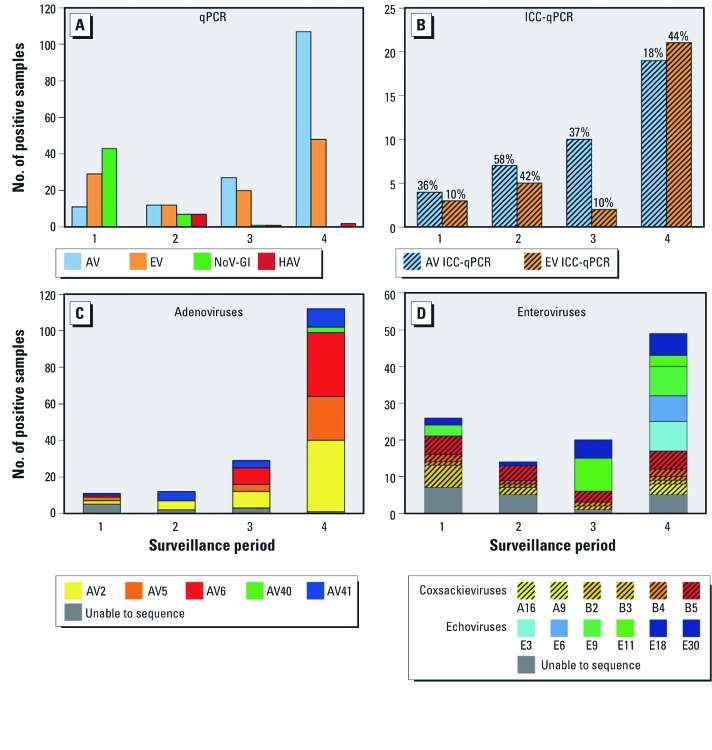
Virus occurrence in tap water by surveillance period. (*A*) Number of virus types detected by qPCR. Abbreviations: AV, adenovirus; EV, enterovirus; NoV-GI, genogroup I norovirus; HAV, hepatitis A virus. (*B*) Number of culturable adenoviruses and enteroviruses determined by ICC-qPCR. Numbers above the bars indicate the percent positive by ICC-qPCR; the denominator, number qPCR-positive, is reported in (*A*). Serotypes and frequency detected of (*C*) adenovirus and (*D*) enterovirus. Of the 157 adenovirus-positive samples and 109 enterovirus-positive samples, 11 (7%) and 18 (17%), respectively, were unable to be sequenced for serotyping.

All 14 study communities had qPCR-measurable viruses in their tap water. Of the 1,204 tap-water samples, 287 (24%) were positive for at least one virus type (Table 1), and 41 (3%) were positive for two or more types (data not shown). The most frequently detected virus types were adenoviruses, enteroviruses, and norovirus genogroup I (NoV-GI) (Table 1). Drinking water concentrations of enteroviruses and NoV-GI were on the order of ones to hundreds of genomic copies per liter; whereas adenoviruses, although more common, had concentrations that were one or two orders of magnitude lower. Exposure to the different virus types via drinking water varied among surveillance periods, particularly for NoV-GI, which was present primarily in the first two surveillance periods (Figure 1A). Cell cultures of the adenovirus and enterovirus qPCR-positive samples never exhibited cytopathic effect. However, when evaluated by ICC-qPCR, culturable adenoviruses and enteroviruses were detected in 25% and 28% of these samples, respectively (Table 1), and virus culturability varied by period (Figure 1B). Five adenovirus serotypes were identified in the qPCR-positive samples (Figure 1C). Enteroviruses were present in all four periods, but the composition of serotypes dramatically shifted from coxsackieviruses in 2006 to primarily echoviruses in 2007 (Figure 1D).

**Table 1:** Virus types, frequencies, and concentrations by qPCR and frequencies of culturable adenovirus and enterovirus by ICC-qPCR for all tap-water samples (n = 1,204).

Virus concentration (genomic copies/L)				
Virus type	No. of qPCR-positive samples (%)	Mean	95th percentilea	Maximum	No. of ICC-qPCR–positive samples (%)b
Adenovirus		157 (13)		0.07		0.3		10		40/157 (25)
Enterovirus		109 (9)		0.8		0.09		851		31/109 (28)
GI norovirus		51 (4)		0.6		0		116		
GII norovirus		0 (0)		0		0		0		
Hepatitis A virus		10 (1)		0.006		0		4		
Rotavirus		1 (0.1)		0.00002		0		0.03		
All viruses		287 (24)c		1.5		2.1		854		
aThe median and 75th percentile concentrations for all sample groups were zero; therefore, the 95th percentile is reported. bICC-qPCR was performed only on qPCR-positive samples. cThis number is less than the sum of virus types because some samples were positive for two or more viruses.										

Of the 1,204 tap-water samples, 86 (7.1%) were collected during times when the communities were conducting short-term chlorination because of routine maintenance or an *E. coli* detection. Twenty of the 86 samples (23%) were virus-positive, mostly for adenovirus and NoV-GI [see Supplemental Material, Table S4 (http://dx.doi.org/10.1289/ehp.1104499)]. We used all qPCR results in the statistical modeling without any contingencies on tests for infectivity or censoring of samples collected during disinfection.

Characteristics of the 580 adult and 1,079 child study participants are reported in Table 2. Enrollment at the end of surveillance stood at 440 households, 413 adults, and 765 children, a drop-out rate of 29%.

**Table 2:** Characteristics of study households and participants at the beginning of surveillance.

Characteristic	n (%)	
Household size (no. of persons)		
2	17	(3)
3	159	(26)
4	246	(40)
5	136	(22)
≥ 6	63	(10)
Residence type		
Single-family home	572	(92)
Apartment or condo	43	(7)
Other	6	(1)
Faucet or plumbing-filtering device		
Yes	73	(12)
No	547	(88)
Don’t know	1	(< 1)
Primary drinking water source		
Municipal	1,546	(93)
Bottled water	58	(3)
Other	1	(< 1)
Missing	54	(3)
Age (years)a		
≤ 2	147	(9)
3–5	277	(17)
6–12	575	(35)
19–30	193	(12)
31–50	440	(27)
> 50	27	(2)
Sex (adults)		
Male	107	(18)
Female	473	(82)
Sex (children)		
Male	524	(49)
Female	555	(51)
Race		
White	1,550	(93)
Nonwhite	96	(5)
Missing	13	(1)
aChildren 13–18 years of age were not eligible for enrollment.		

Over the 48 surveillance weeks, 1,843 AGI episodes and 394,057 person-days of follow-up were recorded [for complete data on AGI episodes and person-time by age group, surveillance period, and community, see Supplemental Material, Table S5 (http://dx.doi.org/10.1289/ehp.1104499)]. AGI incidence over the 48 surveillance weeks by age group was 1.71, 1.78, 1.67, and 2.66 episodes/person-year for all ages, adults, children 6–12 years, and children ≤ 5 years, respectively.

**Figure 2:**
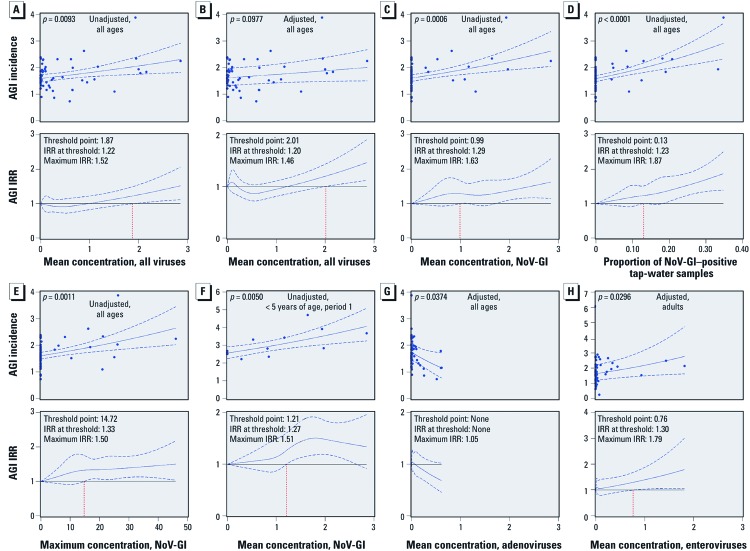
Association between AGI incidence (episodes/person-year) and virus exposure measures in tap water. Top: linear (in the log of the AGI incidence) fits derived from Poisson regression models; each data point represents a community and period. Bottom: AGI incidence rate ratios (IRRs, a measure of relative risk) based on spline fits, with the vertical red dashed line indicating the virus exposure threshold above which AGI risk was significantly elevated. (*A*) All-viruses mean concentration, study participants of all ages, unadjusted. (*B*) All-viruses mean concentration, all ages, adjusted. (*C*) NoV-GI mean concentration, all ages, unadjusted. (*D*) Proportion of NoV-GI–positive samples, all ages, unadjusted. (*E*) Maximum NoV-GI concentration, all ages, unadjusted. (*F*) Surveillance period 1 only, NoV-GI mean concentration, children < 5 years of age, unadjusted. (*G*) Adenovirus mean concentration, all ages, adjusted. (*H*) Enterovirus mean concentration, adults, adjusted. Coefficients for all models are provided in Supplemental Material, Table S3 (http://dx.doi.org/10.1289/ehp.1104499). Blue dashed lines represent 95% CIs; all virus concentrations are expressed as genomic copies per liter. Adjusted models included random intercepts for community and surveillance period.

The mean concentration of all viruses in tap water was associated with AGI incidence (Figure 2A). The AGI IRR (i.e., relative risk) was elevated by 22% [95% confidence interval (CI): 0.04, 49%] when the mean virus concentration exceeded 1.9 genomic copies/L (relative to no viruses present); at the highest mean concentration, AGI IRR increased 52% (95% CI: 12, 106%). Adjusting for underlying differences among communities or surveillance periods, the association strength was diminished (Figure 2B), although AGI IRR was still significantly elevated by 20% (95% CI: 0.04, 44%) at a mean concentration of 2.0 genomic copies/L and the maximum relative risk was increased by 46% (95% CI: 12, 91%) (Figure 2B). Another exposure measure, the maximum concentration of all viruses in tap water, was also associated with AGI incidence in unadjusted (*p* = 0.0044) and adjusted (*p* = 0.0638) models. Complete modeling results for the three virus exposure measures, four virus types, and four age groups are reported in Supplemental Material, Table S3 (http://dx.doi.org/10.1289/ehp.1104499).

All three exposure measures for NoV-GI were associated with AGI incidence (Figure 2C,D,E) and across all age groups. AGI IRR was significantly elevated when the mean concentration, proportion NoV-GI–positive samples, and maximum concentration exceeded 1 genomic copy/L, 13%, and 14.7 genomic copies/L, respectively. The estimated AGI risk was nearly doubled at the highest measured proportion of samples positive for NoV-GI (35%) compared with AGI risk in the absence of NoV-GI (IRR 1.87; 95% CI: 1.39, 2.51%) (Figure 2D). The strengths of associations were reduced in the models adjusted for community and period [see Supplemental Material Table S3 (http://dx.doi.org/10.1289/ehp.1104499)], likely because NoV-GI occurrence was correlated with surveillance period (Figure 1A). To assess the association with AGI when NoV-GI was most abundant in tap water, we conducted two subanalyses restricted to only period 1 for two age groups, all ages and children < 5 years of age; the associations remained positive and were particularly strong for young children (Figure 2F; see also Supplemental Material, Table S3).

Adenovirus exposure measures were not positively associated with AGI, and when significantly associated, the trend was negative [Figure 2G; see also Supplemental Material, Table S3 (http://dx.doi.org/10.1289/ehp.1104499)].

Enteroviruses were not associated with AGI in the unadjusted models. However, in adults when adjusted for community and period, all three enterovirus exposure measures [mean concentration (Figure 2H, *p* = 0.03), proportion positive-samples (*p* = 0.074), and maximum concentration (*p* = 0.028)] were associated with AGI [see Supplemental Material, Table S3 (http://dx.doi.org/10.1289/ehp.1104499)].

One aggregate exposure measure, a mean concentration value for tap-water samples that had unusually high NoV-GI concentrations in one community during period 1 was excluded from analysis. This data point had undue influence on linear model fitting and, when included, the virus concentration–AGI relationship appears asymptotic, similar to the shape of pathogen dose–response curves [see Supplemental Material, Figure S2 (http://dx.doi.org/10.1289/ehp.1104499)]. A second outlier was a single tap-water sample containing 854 genomic copies/L, more than two orders of magnitude higher than the next highest virus concentration measured in that community and period. This single sample outlier was excluded and a new mean concentration value for that community and period was recalculated and incorporated into the modeling analysis.

**Figure 3:**
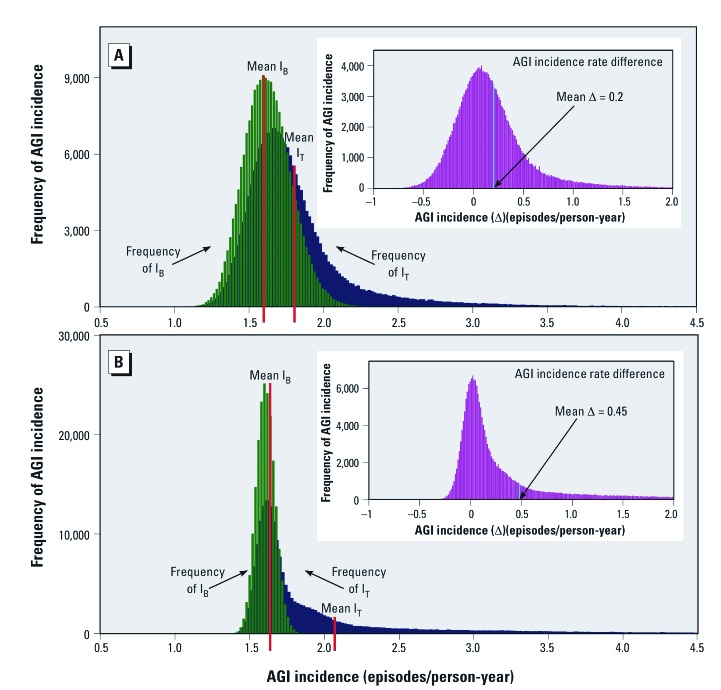
QMRA estimates of the AGI incidence rate difference between viruses absent and viruses present in nondisinfected tap water. Frequency histograms generated by Monte Carlo simulations using two of the virus concentration–AGI incidence models: (A) All viruses, all ages (adjusted model shown in Figure 2B); (B) NoV-GI, all ages (unadjusted model shown in Figure 2C). Green: frequency of baseline AGI incidence (IB) with mean IB [values of IB represent AGI from all sources except viruses in tap water (i.e., unexposed, virus concentrations = 0)]; blue: frequency of total AGI incidence (IT) with mean IT [values of IT represent AGI incidence from all sources including viruses in tap water (i.e., exposed, virus concentrations > 0)]; insets: frequency of AGI incidence rate difference (Δ) between viruses absent and viruses present in nondisinfected tap water. The mean AGI incidence rate difference is indicated by the vertical blue line. Three independent Monte Carlo trials (n = 200,000 for each trial) for both (A) and (B) showed the mean estimates of the incidence rate difference varied maximally by only 0.07% and 0.2%, respectively.

The fraction of AGI attributable to viruses in the nondisinfected drinking water of the communities was estimated using QMRA. To illustrate the potential range in attributable fraction estimates, we used two of the virus exposure–AGI incidence models. Conservatively, using the adjusted model for all-viruses mean concentration, all ages (Figure 2B), the estimated mean incidence rate difference was 0.2 episodes/person-year (Figure 3A). Dividing by the mean total AGI incidence from all transmission routes including tap water (mean I_T_) yields an attributable fraction of 11%. Calculated from the medians of the same frequency distributions, the estimated incidence rate difference and attributable fraction are 0.12 episodes/person-year and 7%, respectively. Repeating the analysis using the unadjusted model for NoV-GI mean concentration, all ages, (Figure 2C), the mean incidence rate difference is 0.45 episodes/person-year (Figure 3B), which corresponds to an attributable fraction of 22%. Derived from the medians, the incidence rate difference is 0.11 episodes/person-year and the attributable fraction is 6%.

## Discussion

qPCR measurements of human enteric viruses in nondisinfected tap water were associated with the incidence of AGI in our study population. There is only one report of a similar finding, the association between qPCR-measured enterococci in recreational water and AGI in swimmers (Wade et al. 2006). As a tool for evaluating infection risk, qPCR is often criticized for being able to detect only pathogen genomes without providing any information on infectivity. Our findings suggest this limitation is not as great as generally believed and that qPCR-positive results for waterborne viruses have value for predicting AGI risk, at least for systems that do not maintain a disinfectant residual. All three virus exposure measures had thresholds above which AGI risk was significantly elevated.

We estimate 6–22% of the AGI in the study communities was attributable to viruses in nondisinfected tap water. These estimates were derived by QMRA using the tap-water virus concentrations measured when UV disinfection was absent. By comparison, consuming drinking water from surface water sources has been estimated to be responsible for 8.5% (Messner et al. 2006) to 12% (Colford et al. 2006) of AGI in immunocompetent populations in developed countries. The long right-side tails of the incidence rate difference frequency distributions (Figure 3 insets) suggests AGI from waterborne viruses in some communities and time periods may have been very high at some point. One such time may have been period 1 when NoV-GI was most abundant in tap water. Restricting the QMRA to period 1 and using the NoV-GI model for period 1, all ages [see Supplemental Material, Table S3 (http://dx.doi.org/10.1289/ehp.1104499)], the fractions of AGI in the communities attributable to viruses in nondisinfected tap water estimated using the means and medians of the frequency distributions were 42% and 23%, respectively. Estimates from the period 1 NoV-GI model for children < 5 years of age suggest that norovirus-contaminated drinking water was responsible for 63% to 44% (calculated from means and medians, respectively) of the AGI in this young age group.

The virus contamination source was likely wastewater from leaking sanitary sewers, a source of urban groundwater contamination (Rutsch et al. 2008). The communities are all served by centralized wastewater treatment, not septic systems, and as is typical of many communities that rely on groundwater, their wells are drilled in established city neighborhoods among the network of sanitary sewer pipes. In a companion study, a subset of the communities’ wells were found to contain wastewater tracers such as ionic detergents, flame retardants, and cholesterol (Hunt et al. 2010).

Two routes were possible for viruses to reach the communities’ tap water: *a*) via virus-contaminated well water pumped into the distribution system, and *b*) direct entry of viruses into the distribution system piping. When UV disinfection was absent, both routes were possible. When UV disinfection was present, the latter route predominated. Only 10% of the well water samples immediately after UV disinfection were virus-positive by qPCR, mostly for adenoviruses [see Supplemental Material, Table S6 (http://dx.doi.org/10.1289/ehp.1104499)], the virus group most resistant to UV disinfection (Yates et al. 2006). As shown in a companion study by Lambertini et al. (2011), the majority of viruses reaching household taps during UV intervention entered the study communities’ distribution systems downstream from UV disinfection, likely from operation and maintenance procedures that directly contaminated pipes or from intrusions through leaks or other types of backflows during transient negative pressure events (LeChevallier et al. 2003).

NoV-GI was the tap-water virus most strongly associated with AGI incidence in the communities. The robustness of the associations may be related to the propensity of norovirus to cause AGI. Most people are susceptible, immunity is short lived, the infectious dose is low (Teunis et al. 2008), and vomiting and diarrhea symptoms can be severe. The measured associations might have been even greater if virus sampling and illness surveillance had been conducted in the winter months when norovirus infections tend to peak (Rohayem 2009).

Adenoviruses were not positively associated with AGI. Two explanations are possible. First, of the five adenovirus serotypes identified in the households’ tap water, only two (serotypes 40 and 41) cause AGI, primarily in infants, and these two serotypes occurred infrequently. The other three detected serotypes primarily cause upper respiratory infections (Robinson and Echavarria 2007). [Even the respiratory adenoviruses are shed in stool (Robinson and Echavarria 2007) and can occur in fecally contaminated water.] Second, adenovirus concentrations were very low compared with norovirus and enterovirus concentrations, and exposure may never have exceeded the threshold necessary to observe elevated AGI. Even for highly infectious NoV-GI, AGI IRR was not significantly elevated until the mean concentration exceeded 1 genomic copy/L, well above the highest adenovirus mean concentration. These explanations may account for the null associations, but the observed negative associations remain perplexing.

Enteroviruses were associated with adult AGI, but only in the adjusted models. The adjustment may have accounted for a period-related shift in the composition of enterovirus serotypes present in the communities’ tap water. In periods 1 and 2, coxsackieviruses constituted 79% of the identified serotypes; whereas in periods 3 and 4, 73% were echoviruses. We explored this hypothesis by restricting the analysis to periods 3 and 4. The association between mean enterovirus concentration and adult AGI was then present also in the unadjusted model [see Supplemental Material, Figure S3 and Table S3 (http://dx.doi.org/10.1289/ehp.1104499)], suggesting the echoviruses were responsible for the observed associations. Enterovirus infectivity varying by period is an alternative explanation for the associations measured in the adjusted models. However, separate analysis of period 2, when coxsackieviruses predominated and the proportion of samples positive for culturable enteorviruses was high (42%), showed no enterovirus–AGI associations. Adult diarrhea from echovirus infection has been reported (Cramblett et al. 1962; Klein et al. 1960), and susceptibility to enterovirus-related illnesses strongly depends on age, with the most severe disease sometimes observed only in adults (Pallansch and Roos 2007).

Several points should be considered when interpreting the data. Participants may have been aware when UV disinfection was installed in their community, affecting their reports of AGI symptoms. Insofar as UV disinfection at the wellheads also affected tap-water virus exposures, it could confound the virus exposure–AGI incidence associations. To test this possibility, we fit the Poisson regression models with and without a dichotomous variable indicating UV disinfection status and compared corresponding IRR estimates. We found no evidence that the associations were confounded by UV disinfection [see Supplemental Material, pp. 35–36 and Table S7 (http://dx.doi.org/10.1289/ehp.1104499)]. Participants were unaware of tap-water virus exposures because these data were not shared. A 12-week surveillance period was selected *a priori* as the least arbitrary and subjective time period for data aggregation. To assess model sensitivity to the data aggregation time period we conducted analyses where outcome and exposure data were aggregated at the calendar month level. Although the outcome and exposure data exhibited substantially more variability, some virus exposure–AGI incidence associations were still observed, particularly for NoV-GI (see Supplemental Material, pp. 35–36 and Table S8). Whether participant drop-out and reporting frequency affected the study findings was evaluated by repeating all modeling with the subset of participants who had completed the entire 48 weeks of follow-up and had missed submitting five or fewer weekly symptom checklists; 1,000 participants met these criteria. While the strength and precision of the associations were generally reduced, the findings were consistent with the full analysis, suggesting reporting bias was not substantial (data not shown). Selection bias could still exist if those agreeing to participate in the study were not representative of AGI susceptibility in their community. Lastly, the qPCR results likely underestimate the true virus quantities because of virus losses during sample filtration, secondary concentration, and nucleic acid extraction steps.

The drinking water sanitary quality of the 14 study communities, as indicated by the detection frequency of total coliform bacteria, was similar to other untreated groundwater sources in the United States (data not shown). Using the U.S. EPA total coliform data and occurrence model (U.S. EPA 2006c), the maximum likelihood estimate of the national average total coliform detection rate for routine samples from small community water systems (population ≤ 4,100) that use untreated groundwater is 2.4%. The average rate among the 14 systems during the study was 2.3%. Examining the 14 systems individually, they lie between the 28th and 81st percentiles in the maximum likelihood beta distribution of total coliform detects, suggesting that none of the study communities could be considered outliers with respect to drinking water sanitary quality.

Groundwater provides drinking water for 114 million Americans (U.S. EPA 2006b). Unlike surface water sources for drinking water, little federal regulatory attention had been given to groundwater. That changed with the Groundwater Rule (U.S. EPA 2006a), which emphasizes a risk-based strategy using sanitary surveys and well water monitoring for fecal indicators to identify groundwater supplies vulnerable to fecal contamination. The rule does not require disinfection, but if disinfection is deemed necessary, the technology selected must reduce well water virus concentrations by 99.99% (U.S. EPA 2006a). Groundwater-borne viruses are also receiving attention in the proposed third Unregulated Contaminant Monitoring Rule, which would authorize national monitoring for enterovirus and norovirus in nondisinfecting public water system wells (U.S. EPA 2011). Our study findings suggest that protecting aquifer sanitary quality and ensuring the water is adequately disinfected would be significant steps towards reducing the AGI burden from virus-contaminated groundwater.

## Supplemental Material

(1.2 MB) PDF
