# Supplementary material for: Viruses in Nondisinfected Drinking Water from Municipal Wells and Community Incidence of Acute Gastrointestinal Illness
Source: Environ Health Perspect. 2012 Jun 1;120(9):1272–9. doi: 10.1289/ehp.1104499 (PMC3440111; doi:10.1289/ehp.1104499)
Supplement: (1.2 MB) PDF [file ehp.1104499.s001.pdf]

Supplemental Material

Viruses in Non-Disinfected Drinking Water from Municipal Wells and Community Incidence of Acute Gastrointestinal Illness

Mark A. Borchardt[1,*], Susan K. Spencer[1,*], Burney A. Kieke Jr. [1],
Elisabetta Lambertini[2], and Frank J. Loge[2]

[1]Marshfield Clinic Research Foundation, Marshfield, WI 54449, USA
[2]Department of Civil and Environmental Engineering, University of California, Davis, CA 95616, USA

[*]Current address: USDA – Agricultural Research Service, Marshfield, WI 54449

Address correspondence to Mark A. Borchardt, USDA-Agricultural Research Service, 2615 Yellowstone Dr. Marshfield, WI 54449 Telephone: 715-387-4943, Fax: 715-384-9157, E-mail: mark.borchardt@ars.usda.gov

Table of Contents

Supplemental Material Figure S1

Recruitment flow chart of the numbers of households and participants…………..…….4

Laboratory Materials and Methods

Virus sampling controls ……………………………………………………………..…5

Nucleic acid extraction ……………………………………………………………..….5

Reverse transcription (RT)……………………………………………………….….…5

qPCR…………………………………………………………………………………....6

qPCR controls……………………………………………………………………….....6

Supplemental Material Table S1

Primers and TaqMan probes for human enteric virus detection by qPCR………………..7

Laboratory Materials and Methods

Standard curves for qPCR………………………..…………………………………....9

Supplemental Material Table S2

qPCR standard curves quality assurance parameters……………………………………11

Laboratory Materials and Methods

Inhibition control……………………………………………………………………...12

Virus concentration calculation……………………………………………………..…12

Adenovirus and enterovirus serotyping…………………………………………….…13

Adenovirus and enterovirus cell culture………………………………………………13

Statistical Models Interpretation ………………………..………………………………………14

Quantitative Microbial Risk Assessment…………………….………………………………..15

Supplemental Material Table S3

Poisson regression modeling results……………………………………………………..18

Supplemental Material Table S4

Virus results for samples collected during chlorination…………………………………22

Supplemental Material Table S5

AGI episodes and person-time by age group, surveillance period, and community …....23

Supplemental Material Figure S2

Spline fit depicting the influence of an outlier…………………………………………...32

Table of Contents (cont)

Supplemental Material Table S6
Virus results for well water samples following UV disinfection……………..…………33

Supplemental Material Figure S3
Enterovirus mean concentration and AGI incidence, periods 3 and 4...…………………34

Statistical Models Sensitivity Analyses ………………………………………………………35

Supplemental Material Table S7
Examination of potential confounding by UV disinfection status ………………………37

Supplemental Material Table S8
Poisson regressions with data aggregated at the level of calendar month ………………38

References……………………………………………………………………………………..40

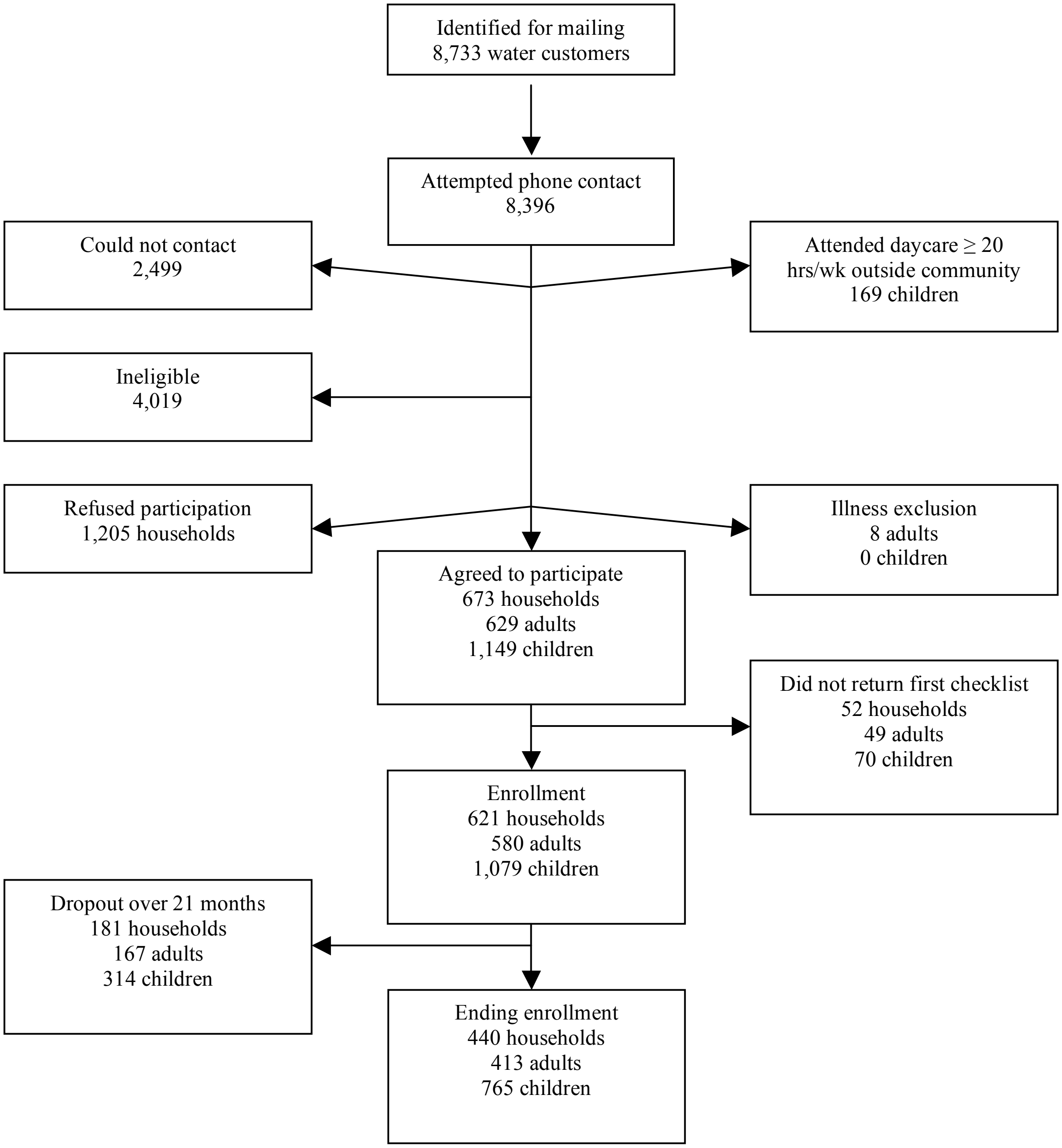

Supplemental Material Figure S1. Flow chart of the number of households and participants during the recruitment process. Water customers include all service connections, not just households.

Laboratory Materials and Methods

Virus sampling controls. Equipment blank controls were performed once per surveillance period; all were negative for the six virus types investigated in this study. After every preparation of washed glass wool a blank filter was constructed and analyzed as if it were an unknown sample; all glass wool preparations were virus negative. Glass wool performance controls were performed once per surveillance period. Ten liters dechlorinated laboratory tap water was seeded with 1×10^4 genomic copies poliovirus Sabin type 3 and recovery was performed as described in Lambertini et al. (2008). Recovery efficiencies ranged from 70% to 96%. Water matrix recovery controls were performed once for each of the 14 study communities using 2 to 4 replicate 10-liter water volumes. Recovery efficiencies among the 14 communities ranged between 23% and 99%.

Nucleic acid extraction. Nucleic acids from both RNA and DNA viruses were extracted from 280 μL of final concentrated sample volume (FCSV) with the QIAamp DNA blood mini kit and buffer AVL (Qiagen, Valencia, CA). Final volume of the nucleic acid suspension was 50 μL.

Reverse transcription (RT). RNA viruses were reverse-transcribed by adding 11.18 μL nuclease-free water and 0.91 μL random hexamers (ProMega, Madison, WI) to 11.18 μL of the extracted nucleic acids. This mixture was heated for 4 min at 99°C and then mixed with 41.73 μL RT master mix consisting of the following components reported as final concentrations in the 65 μL total reaction volume: 10 mM Tris-HCl (pH 8.3), 50 mM KCl, 3 mM $MgCl_2$, 10 mM dithiothreitol, 70 μM concentration of each deoxynucleoside triphosphate (ProMega), 30 U RNAsin (ProMega), 100 U SuperScript II reverse transcriptase (Invitrogen Life Technologies, Rockville, MD). Reaction incubation was at 25°C for 15 min, 42°C for 60 min, and 99°C for 5 min and then held at 4°C until PCR amplification the same day.

qPCR. The 20 µL final reaction volume consisted of 14 µL master mix to which was added 6 µL extracted DNA (adenoviruses) or cDNA from the RT step. Primers (Integrated DNA Technology, Coralville, IA) and TaqMan probes (TIB Molbiol, Berlin, Germany) and their concentrations are reported in Supplemental Material, Table S1. Thermocycling began with 95° C for 10 min followed by 45 cycles of 15 s at 94° and 1 min at 60° C.

qPCR controls. Every batch of PCR reactions included the following negative controls: 1) Negative extraction control, which was FCSV created from a blank filter using the same elution and secondary concentration steps as a real sample; 2) Negative RT master mix; and 3) Negative PCR master mix. If any of the negative controls were positive the data were omitted, the source of the contamination identified and corrected, and the analysis batch repeated.

Every batch of PCR reactions included the following positive controls: 1) Positive extraction control, which was the same as the enterovirus reference control seeded into "blank" FCSV matrix; and 2) Positive reference control for each virus group tested. The standard of each virus that resulted in a crossing point of near 34 was aliquoted and stored frozen to be used subsequently as the reference control. Reference controls for noroviruses GI and GII, rotavirus, and HAV were in the form of cDNA, the reference control for adenovirus was extracted DNA, and the enterovirus reference control was intact virus because this control also served as the nucleic acid extraction positive control for the entire analysis batch. New reference controls were created at the same time as the standard curves. Positive reference controls were required to be within ± 0.5 cycles of the original crossing point measured when the standard curve was created in order for the measurements of the unknown samples to be acceptable. An analysis batch was repeated if the reference control fell outside this range.

Supplemental Material Table S1. Primers and TaqMan probes for human enteric virus detection by qPCR. Primer final concentrations (nM) in master mix are noted in parentheses. Probe final concentrations are all 100 nM.

Virus group	Primer pairs	TaqMan Probe	Reference[a]
Adenovirus	GGACGCCTCGGAGTACCTGA (500)<br>CGCTGIGACCIGTCTGTGG (500)	CACCGATACGTACTTCAGCCTGGGT	1
Enterovirus	CCTCCGGCCCCTGAATG (300)<br>ACCGGATGGCCAATCCAA (900)	CGGAACCGACTACTTTGGGTGTCCGT	2, 3
GI Norovirus	GCCATGTTCCGITGGATG (500)<br>TCCTTAGACGCCATCATCAT (500)	TGTGGACAGGAGATCGCAATCTC	4
GII Norovirus	TGGAATTCCATCGCCCACTGG (250)<br>TGTCACGATCTCATCATCACC (250)	ATGTCAGGGGACAGGTTTGT<br>ATGTCGGGGCCTAGTCCTGT	5
Hepatitis A	CTCCAGAATCATCTCCAA (700)<br>CAGCACATCAGAAAGGTGAG (700)	AATGTTTATCTTTCAGCAATTAATCTGGA	6
Rotavirus A	TTGCCACCAATTCAGAATAC (500)<br>ATTTCGGACCATTTATAACC (500)	ACAGTATAAGAGAGCACAAGTTAATGAAACA	7
Hepatitis G	CGGCCAAAAGGTGGTGGATG (500)<br>CGACGAGCCTGACGTCGGG (500)	AGGTCCCTCTGGCGCTTGTGGCGAG	8

[a] References for primers and probes

1. Cromeans T, Narayanan J, Jung K, Ko G, Wait D, Sobsey M. 2005. Development of molecular methods to detect infectious viruses in water. Report No. 90995F. American Water Works Association Research Foundation (AwwaRF), Denver, CO.

2. De Leon R, Shieh C, Baric RS, Sobsey MD (1990) Detection of enterovirus and hepatitis A virus in environmental samples by gene probes and polymerase chain reaction. Proceedings of the American Water Works Association Water Quality and Technology Conference, Denver, CO, p. 833-853.

3. Monpoeho S, Dehee A, Mignotte B, Schwartzbrod L, Marechal V, Nicolas JC, Billaudel S, Ferre V. 2000. Quantification of enterovirus RNA in sludge samples using single tube real-Time RT-PCR. Biotechniques 29:88-93.

4. Jothikumar N, Lowther JA, Henshilwood K, Lees DN, Hill VR, Vinje J. 2005. Rapid and sensitive detection of noroviruses by using TaqMan-based one-step reverse transcription-PCR assays and application to naturally contaminated shellfish samples. Appl Environ Microbiol 71:1870-1875.

5. Ando T, Monroe SS, Gentsch JR, Jin Q, Lewis DC, Glass RI. 1995. Detection and differentiation of antigenically distinct small round-structured viruses (Norwalk-like viruses) by reverse transcription-PCR and southern hybridization. J Clin Microbiol 33:64-71.

6. Schwab KJ, DeLeon R, Sobsey MD. 1995. Concentration and purification of beef extract mock eluates from water samples for the detection of enteroviruses, hepatitis A virus and Norwalk virus by reverse transcription PCR. Appl Environ Microbiol 61:531-537.

7. Gentsch JR, Glass RI, Woods P, Gouvea V, Gorziglia M, Flores J, Das BK, Bhan MK. 1992. Identification of group A rotavirus gene 4 types by polymerase chain reaction. J Clin Microbiol 30:1365-1373.

8. Schlueter V, Schmolke S, Stark K, Hess G, Ofenlock-Haehnle B, Engel AM. 1996. Reverse transcription-PCR detection of hepatitis G virus. J Clin Microbiol 34:2660-2664.

Standard curves for qPCR. Stocks of each of the virus groups were used to generate the qPCR standard curves. Adenovirus stock was derived from A549 cell cultures of adenovirus 41, and enterovirus stock was derived from BGM cell cultures of poliovirus attenuated vaccine strain Sabin type 3. After cytopathic effect was observed, the viruses were released by freeze-thawing the infected cell monolayers three times followed by removal of cell debris by centrifuging at 900 x *g* for 10 min. Norovirus genogroups I and II and rotavirus group A were purified from human stool specimens by diluting stool 1:2 with sterile PBS, adding this to Vertrel XF (Miller-Stephenson, Sylmar, CA, USA) to create a 25% suspension, and separated by centrifugation at 2,100 x *g* for 10 min. HAV was purchased as Armored RNA (Asuragen Inc., Austin, TX). All virus preparations were stored at -80°C.

Extraneous nucleic acids in the virus stock preparations (except for HAV Armored RNA) was removed by treatment with Benzonase (Novagen, Madison, WI) for 30 min at 37°C followed by incubation for 2 days at 4°C. This method leaves behind only the nucleic acid protected by intact viral capsids so that when it is subsequently extracted and quantified the nucleic acid accurately reflects the actual number of virions.

After Benzonase treatment, viral nucleic acid was extracted using the QIAamp DNA blood mini kit, but without adding carrier RNA to the AVL buffer as this extra RNA would have inflated the apparent virus copy number.

Viral DNA or RNA mass was measured using a CytoFluor series-4000 fluorimeter (Applied Biosystems, Framington, MA) and the DNA or RNA intercalating dyes PicoGreen or RiboGreen, respectively, (Molecular Probes, Eugene, OR). Nucleic acid mass was converted to genomic copies using the nucleic acid molecular weight for each virus (Roche Molecular Biochemicals 2000).

Standards were created by serially diluting 1:10 the capsid-intact virions (now Benzonase treated and quantified) into 280 µL volumes of negative FCSV. Negative FCSV was prepared by passing 10 L dechlorinated tap water through a glass wool filter and eluting and flocculating the eluate the same as for the unknowns. Each 1:10 dilution was independently extracted with the QIAamp DNA blood mini kit and the virus quantified following the reverse transcription and qPCR procedures as for the unknowns. This approach for generating qPCR standard curves encompasses the entire quantitation process and includes any matrix effects from the filter elution and secondary concentration procedures. Crossing points (Cp) were calculated using the second-derivative-maximum method and regressed against the decimal logarithm of virus copy number using the non-linear function provided by the LightCycler 480 instrument.

A new standard curve for each virus group was generated approximately every four months, just prior to analyzing the water samples that had been collected during the previous surveillance period. Quality assurance parameters for the standard curves are reported in Supplemental Material, Table S2.

Supplemental Material Table S2. qPCR standard curves quality assurance parameters.				
Virus	Standard Curve #	Efficiency	r^2	Highest Cp Standard Measured
Adenovirus	1	1.940	0.978	38.44
	2	2.094	0.971	38.10
	3	1.940	0.987	37.94
	4	1.915	0.992	38.33
	5	1.960	0.976	38.10
Enterovirus	1	2.159	0.958	40.00
	2	2.252	0.986	37.05
	3	1.949	0.999	38.26
	4	1.925	0.984	36.72
	5	1.943	0.973	40.00
GI Norovirus	1	1.905	0.962	37.02
	2	1.928	0.959	37.08
	3	1.978	0.980	40.00
	4	2.044	0.975	40.00
	5	1.958	0.997	37.29
GII Norovirus	1	1.909	0.962	39.55
	2	1.858	0.979	40.00
	3	1.949	0.985	37.24
	4	2.029	0.943	37.76
	5	1.968	0.974	38.29
Hepatitis A	1	2.131	0.993	40.00
	2	2.031	0.957	38.71
	3	2.266	0.990	37.59
	4	2.033	0.984	38.39
	5	1.995	0.975	40.00
Rotavirus	1	1.955	0.985	40.00
	2	2.076	0.987	40.00
	3	1.896	0.996	37.46
	4	1.940	0.978	36.82
	5	1.960	0.958	40.00

Inhibition control. RT-qPCR inhibition was evaluated for every unknown sample by spiking hepatitis G virus armored RNA (HGV) (Asuragen Inc., Austin, TX) into the RT reaction mixture and performing RT-qPCR as described for the other viruses. The target HGV concentration in the qPCR reaction was a crossing point (Cp) of 30. HGV primers and probe are reported in Supplemental Material, Table S1. A sample was deemed uninhibited if the HGV Cp was no more than one cycle higher than the expected Cp for the seeded HGV. If the sample was inhibited (i.e., > 1 cycle difference) the difference in crossing points was used to calculate an appropriate dilution with nuclease-free water as follows:

Dilution factor = 10^x,

where x = (Expected HGV Cp – Measured HGV Cp) ÷ standard curve slope

For example, if x = 0.845 the dilution factor = 7 and the nucleic acid extraction was diluted 1:7 with nuclease-free water before adding it to the RT master mix.

Virus concentration calculation. Final virus concentrations were calculated with the following equation. Steps in the equation correspond to procedural steps that result in a proportion or multiplier that is necessary for calculating the final virus concentration.

Step 1: Number genomic copies measured in PCR reaction

Step 2: ÷ $\frac{\text{volume of RT reaction added to the PCR reaction}}{\text{RT reaction volume}}$

Step 3: ÷ $\frac{\text{volume of nucleic acid extraction added to the RT reaction}}{\text{nucleic acid extraction volume}}$

Step 4: ÷ $\frac{\text{volume of FCSV extracted}}{\text{FCSV volume}}$

Step 5: x dilution factor to mitigate inhibition (factor = 1 if no inhibition)

Step 6: ÷ water sample volume filtered in liters

Step 7: = Number virus genomic copies/liter

Adenovirus and enterovirus serotyping. All enteroviruses and adenoviruses in qPCR-positive samples were serotyped by nucleotide sequencing. For enteroviruses, a separate PCR targeting a 656 base pair region encoding one-third of the 5' UTR (untranslated region), the entire VP4 region, and one-third of VP2 was performed using primers OL68-1 and EVP4 (Ishiko et al. 2002). For adenoviruses, the 263 bp product from the qPCR that targeted the hexon gene was sequenced. Amplified DNA was visualized by gel electrophoresis and purified with the Qiaquick PCR Purification Kit. (Qiagen, Valencia, CA). Sequencing was conducted in both directions with the BigDye Terminator Cycle Sequencing Kit (Applied Biosystems, Foster City, CA) and the ABI Prism 3100 Gene Analyzer. Consensus sequences were constructed with Lasergene (DNAStar, Madison, WI). Sequences were submitted for identification using BLAST (National Center for Biotechnology Information, Bethesda, MD).

Adenovirus and enterovirus cell culture. Enterovirus-qPCR positive samples were inoculated into three cell lines: Buffalo green monkey kidney (BGM), rhabdomyosarcoma (RD), and human colonic carcinoma cells (CaCo-2). Adenovirus-qPCR positive samples were inoculated into two cell lines: human embryo kidney cells (Graham 293) and human lung adenocarcinoma epithelial cells (A549). Cells were grown to 60% to 80% confluence in 25 cm^2 tissue culture flasks with Eagle minimal essential medium with Earle's salts, HEPES buffer, penicillin-streptomycin-fungizone solution and 10% fetal bovine serum. Final concentrated sample (800 µL FCSV) was added to 3.2 mL sterile PBS and then passed through a 0.22 µm pore size sterilizing filter (Acrodisc, Pall Life Sciences, Port Washington, NY, USA). Cell cultures were inoculated by pouring off the growth media, rinsing with sterile PBS, and adding 500 µL of the FCSV solution to each of the three (enteroviruses) or two (adenoviruses) cell lines. Flasks were rocked for 90 min at room temperature, the inoculum was decanted, the cell layer

was washed with pre-warmed PBS containing 2% fetal bovine serum, and then 10 ml of Eagle minimal essential medium with 2% fetal bovine serum was added to each flask. Incubation was at 37°C; inoculated cell cultures were replenished with fresh maintenance media every seven days. Each set of inoculated flasks included a sterile PBS negative control and positive controls inoculated with poliovirus or adenovirus 41. Cultures were examined with an inverted microscope for the appearance of cytopathic effects (CPE) daily for three days and then every other day for two weeks. Cultures that were CPE negative after two weeks, after removing the maintenance media and adding 1 ml sterile H_2O, were freeze-thawed three times to release any potentially present virus. The freeze-thaw lysates (0.2 mL) plus sterile 2x PBS (0.2 mL) was passed into a new 25 cm^2 flask containing the same cell line (60% to 80% confluent) and observed for another two week period. After four weeks, if still CPE negative, a third passage was performed and the culture was observed for two more weeks. All cultures of water samples in this study were passaged three times and observed for six weeks.

Statistical Models Interpretation

All Poisson regression modeling results are reported in Supplemental Material, Table S3. The interpretation of the fixed virus effect differs somewhat for the unadjusted versus adjusted models. The unadjusted models are known as marginal or population-average models (Kaufman 2008). Corresponding virus effects are the average effect pooled over communities and reporting periods. Inference is limited to the communities and reporting periods within the current study. The random intercept adjustment implies that the 14 study communities and four time periods are random samples from populations of similar communities and time periods. This permits inference to similar communities and time periods with different underlying levels of AGI

incidence than those in the present study. The adjusted models are referred to as subject-specific or cluster-specific models (Kaufman 2008). The virus effect is that of a 'typical' or 'average' community and reporting period from the relevant populations, where 'typical' or 'average' is operationalized by setting the random intercepts to their mean value of zero. We opted not to adjust for multiple comparisons in the analyses. Rather, our approach was to evaluate each association in the context of available relevant information (Savitz and Olshan 1995).

Certain assumptions are necessary to generalize the models presented here to estimate AGI risk from qPCR-measured virus levels in other environmental settings. Foremost is the sampling timeframe for characterizing virus exposure. In a new setting the sampling timeframe from which these measures are determined must be assumed to be no different than the 12-week aggregate exposure measures used to construct the models. A second key assumption is that the sampling, secondary concentration, nucleic acid extraction, and qPCR methods used to measure viruses in another setting would yield the same virus concentrations and detection frequencies as obtained in the present study. Another consideration is the qPCR virus measurements in the present study were from non-chlorinating systems. Therefore any condition that would completely inactivate a virus while leaving its nucleic acid amplifiable would result in the models overestimating AGI incidence. Other assumptions are no different than those necessary for extending a dose-response relationship obtained from a human feeding trial to a QMRA for a different population and location.

Quantitative Microbial Risk Assessment

The following steps were carried out for each iteration of the Monte Carlo simulations:

1) N single-sample virus concentration values were randomly selected from the data set of tap water samples collected during the periods when the UV disinfection intervention was absent from a community (number of samples in the data set = 618). The QMRA conducted with only the period 1 tap water data included 136 samples in the data set. N was also randomly selected at each iteration to be within the range of the actual number of tap water samples collected from a study community during a 12-week period (between 17 and 24 samples, uniform distribution). The data set included both zero and non-zero values, empirically representing the temporal and spatial variability in virus contamination observed during the study;
2) The arithmetic mean of the N concentration values was calculated to obtain a 12-week mean virus concentration, consistent with the level of time aggregation used as the predictor variable in the virus exposure – AGI response models;
3) The mean concentration was input into the exposure-response relationship (Eq. 1), along with an error term randomly drawn from a normal distribution with a mean of zero and variance σ^2 (Eq.2). Model coefficients and corresponding variance/covariance estimates are reported in Supplemental Material, Table S3. The output was a realization of the total AGI incidence (I_T) attributable to tap waterborne viruses plus other sources and expressed as number of AGI episodes/person-yr.

$$\text{AGI Incidence} = 365.25*\exp(\text{intercept}+\text{beta}*\text{Concentration}+\text{error}) \qquad \text{(Eq.1)}$$

where the error term is $\sim N(0,\sigma^2)$, with

$$\sigma^2=\text{Var(intercept)}+\text{Concentration}^2*\text{Var(beta)}+2*\text{Concentration}*\text{Covar(intercept,beta)} \qquad \text{(Eq. 2)}$$

4) To obtain a realization of the baseline AGI incidence from other sources (I_B), not related to drinking water contamination, a concentration value of zero was input into the exposure-response relationship (Eq. 1), along, again, with a random error term (Eq. 2).

5) To obtain a realization of the AGI incidence rate difference (Δ) when viruses were absent compared with viruses present in non-disinfected drinking water, the baseline incidence estimated in step 4 was subtracted from the total incidence estimated in step 3;

Steps 1-5 were repeated 2 x 10^5 times to obtain the frequency distribution of the AGI incidence rate difference from tap waterborne viruses (i.e., $\Delta_i = (I_T - I_B)_i$ where i = 1…N and N = number of Monte Carlo iterations). The simulation was carried out in MATLAB® R2011a.

Supplemental Material Table S3. Poisson regression modeling results. Regression coefficients and corresponding variance/covariance estimates from the linear (in the log of the AGI incidence) fits and incidence rate ratio (IRR) (i.e., (relative risk) information from the spline fits for each model by participant age group, virus type, and virus exposure measure.
Coefficients are for daily AGI incidence, i.e., AGI episodes/person-day = $e^{(\text{intercept + beta * virus exposure measure})}$. Multiply by 365.25 for annual incidence.

			Linear Fit [a]						Spline Fit [a]		
Participant Age Group	Virus Type	Virus Exposure Measure	P-value for Beta	Beta	Beta Variance	Intercept	Intercept Variance	Beta-Intercept Covariance	Threshold Point for Significant IRR	IRR at Significant Virus Threshold Point	Max IRR [b]
**Unadjusted Model**											
All ages	All	Maximum	0.0044	9.650E-03	1.000E-05	-5.4354	1.873E-03	-7.400E-05	25.581	1.21802	1.55
All ages	All	Mean	0.0093	1.297E-01	2.310E-03	-5.4359	2.005E-03	-1.194E-03	1.8742	1.22256	1.52
All ages	All	Prop pos	0.7434	-8.166E-02	6.160E-02	-5.3558	4.535E-03	-1.333E-02	0.2556	1.25945	1.33
All ages	Adenovirus	Maximum	0.0433	-7.763E-02	1.410E-03	-5.3382	1.735E-03	-5.720E-04			1.02
All ages	Adenovirus	Mean	0.016	-8.662E-01	1.210E-01	-5.3324	1.668E-03	-5.029E-03			1
All ages	Adenovirus	Prop pos	0.0106	-7.350E-01	7.692E-02	-5.2939	2.214E-03	-7.784E-03			1.04
All ages	Enterovirus	Maximum	0.7755	2.730E-03	9.000E-05	-5.3777	1.863E-03	-1.400E-04			1.11
All ages	Enterovirus	Mean	0.81	3.145E-02	1.695E-02	-5.3771	1.872E-03	-1.955E-03			1.11
All ages	Enterovirus	Prop pos	0.9955	2.680E-03	2.213E-01	-5.3738	3.349E-03	-1.942E-02			1.01
All ages	GI Norovirus	Maximum	0.0011	1.083E-02	1.000E-05	-5.4242	1.570E-03	-5.300E-05	14.7229	1.32648	1.5
All ages	GI Norovirus	Mean	0.0006	1.723E-01	2.250E-03	-5.4271	1.543E-03	-7.980E-04	0.9851	1.29488	1.63
All ages	GI Norovirus	Prop pos	<.0001	1.752E+00	1.290E-01	-5.4399	1.339E-03	-5.729E-03	0.126	1.22955	1.87
All ages	GI Norovirus Period 1 only	Mean	0.1864	8.960E-02	4.042E-03	-5.1964	7.597E-03	-3.750E-03			1.36
Adults	All	Maximum	0.0007	1.605E-02	2.000E-05	-5.4450	4.104E-03	-1.540E-04	15.8271	1.32757	2.05
Adults	All	Mean	0.0011	2.281E-01	4.380E-03	-5.4521	4.358E-03	-2.498E-03	1.3428	1.3143	2.05
Adults	All	Prop pos	0.7957	9.853E-02	1.433E-01	-5.3596	1.086E-02	-3.157E-02	0.2567	1.44055	1.54
Adults	Adenovirus	Maximum	0.3052	-6.005E-02	3.360E-03	-5.3105	4.441E-03	-1.410E-03			1
Adults	Adenovirus	Mean	0.1882	-7.383E-01	3.067E-01	-5.3029	4.369E-03	-1.303E-02			1
Adults	Adenovirus	Prop pos	0.0351	-9.733E-01	2.024E-01	-5.2350	5.403E-03	-1.958E-02			1
Adults	Enterovirus	Maximum	0.4151	1.114E-02	1.800E-04	-5.3556	4.468E-03	-3.090E-04			1.23
Adults	Enterovirus	Mean	0.3991	1.597E-01	3.529E-02	-5.3569	4.511E-03	-4.465E-03			1.22
Adults	Enterovirus	Prop pos	0.6958	2.842E-01	5.225E-01	-5.3632	8.166E-03	-4.676E-02			1.08
Adults	GI Norovirus	Maximum	0.0007	1.602E-02	2.000E-05	-5.4163	3.580E-03	-1.170E-04	15.551	1.46442	1.78
Adults	GI Norovirus	Mean	0.0003	2.557E-01	4.470E-03	-5.4214	3.487E-03	-1.770E-03	0.9908	1.4444	1.97
Adults	GI Norovirus	Prop pos	<.0001	2.639E+00	2.302E-01	-5.4450	2.847E-03	-1.183E-02	0.1315	1.33525	2.61

Supplemental Table S3 (cont)

			Linear Fit [a]						Spline Fit [a]		
Participant Age Group	Virus Type	Virus Exposure Measure	P-value for Beta	Beta	Beta Variance	Intercept	Intercept Variance	Beta-Intercept Covariance	Threshold Point for Significant IRR	IRR at Significant Virus Threshold Point	Max IRR [b]
Adults	Enterovirus Periods 3&4 only	Mean	0.0462	3.374E-01	2.596E-02	-5.5736	4.145E-03	-4.510E-03	0.78	1.43	1.85
Children ≤ 12	All	Maximum	0.072	5.630E-03	1.000E-05	-5.4289	1.528E-03	-6.200E-05			1.31
Children ≤ 12	All	Mean	0.1383	6.800E-02	2.040E-03	-5.4255	1.624E-03	-9.930E-04			1.26
Children ≤ 12	All	Prop pos	0.3812	-1.873E-01	4.497E-02	-5.3532	3.254E-03	-9.627E-03			1.22
Children ≤ 12	Adenovirus	Maximum	0.0074	-8.803E-02	1.000E-03	-5.3538	1.185E-03	-3.990E-04			1.06
Children ≤ 12	Adenovirus	Mean	0.002	-9.398E-01	8.320E-02	-5.3492	1.124E-03	-3.412E-03			1.03
Children ≤ 12	Adenovirus	Prop pos	0.0133	-6.037E-01	5.560E-02	-5.3274	1.669E-03	-5.771E-03			1.1
Children ≤ 12	Enterovirus	Maximum	0.7454	-2.800E-03	7.000E-05	-5.3896	1.350E-03	-1.070E-04			1.04
Children ≤ 12	Enterovirus	Mean	0.6624	-5.085E-02	1.341E-02	-5.3881	1.349E-03	-1.460E-03			1.05
Children ≤ 12	Enterovirus	Prop pos	0.6883	-1.627E-01	1.627E-01	-5.3795	2.417E-03	-1.411E-02			1.01
Children ≤ 12	GI Norovirus	Maximum	0.0129	7.620E-03	1.000E-05	-5.4283	1.281E-03	-4.400E-05	16.5632	1.24123	1.36
Children ≤ 12	GI Norovirus	Mean	0.0098	1.205E-01	2.020E-03	-5.4300	1.277E-03	-6.680E-04	2.0214	1.23029	1.45
Children ≤ 12	GI Norovirus	Prop pos	0.002	1.183E+00	1.318E-01	-5.4366	1.217E-03	-5.320E-03	0.2123	1.27425	1.5
Children < 5	All	Maximum	0.0664	9.390E-03	3.000E-05	-4.9875	3.794E-03	-1.620E-04			1.64
Children < 5	All	Mean	0.0873	1.245E-01	5.110E-03	-4.9869	3.915E-03	-2.431E-03			1.47
Children < 5	All	Prop pos	0.5495	1.940E-01	1.037E-01	-4.9719	7.434E-03	-2.181E-02			1.3
Children < 5	Adenovirus	Maximum	0.6261	-2.421E-02	2.440E-03	-4.9219	3.230E-03	-9.470E-04			1.22
Children < 5	Adenovirus	Mean	0.2446	-5.764E-01	2.400E-01	-4.9079	3.143E-03	-8.985E-03			1.18
Children < 5	Adenovirus	Prop pos	0.6086	-1.973E-01	1.467E-01	-4.9114	4.355E-03	-1.477E-02	0.1319	1.29073	1.37
Children < 5	Enterovirus	Maximum	0.5918	-7.200E-03	1.800E-04	-4.9215	3.165E-03	-2.400E-04			1.05
Children < 5	Enterovirus	Mean	0.6321	-9.009E-02	3.501E-02	-4.9225	3.181E-03	-3.411E-03			1.04
Children < 5	Enterovirus	Prop pos	0.9641	2.843E-02	3.962E-01	-4.9340	5.603E-03	-3.290E-02			1.04
Children < 5	GI Norovirus	Maximum	0.0171	1.215E-02	2.000E-05	-4.9851	3.274E-03	-1.220E-04	18.4036	1.34421	1.55
Children < 5	GI Norovirus	Mean	0.0165	1.826E-01	5.430E-03	-4.9855	3.271E-03	-1.826E-03	1.2242	1.42726	1.59
Children < 5	GI Norovirus	Prop pos	0.0571	1.110E+00	3.258E-01	-4.9744	3.338E-03	-1.396E-02			1.36
Children < 5	GI Norovirus Period 1 only	Mean	0.0050	1.624E-01	2.160E-03	-4.9575	3.465E-03	-1.861E-03	1.21	1.27	1.51

Supplemental Table S3 (cont)												
			Linear Fit [a]						Spline Fit [a]		
Participant Age Group	Virus Type	Virus Exposure Measure	P-value for Beta	Beta	Beta Variance	Intercept	Intercept Variance	Beta-Intercept Covariance	Threshold Point for Significant IRR	IRR at Significant Virus Threshold Point	Max IRR [b]
**Model Adjusted for Community and Period [c]**											
All ages	All	Maximum	0.0638	5.900E-03	1.000E-05	-5.4206	9.243E-03	-6.200E-05	30.918	1.22862	1.49
All ages	All	Mean	0.0977	7.534E-02	1.970E-03	-5.4195	9.720E-03	-9.650E-04	2.0056	1.19839	1.46
All ages	All	Prop pos	0.3826	2.127E-01	5.797E-02	-5.4338	1.557E-02	-1.344E-02	0.2486	1.20772	1.27
All ages	Adenovirus	Maximum	0.0995	-5.231E-02	9.600E-04	-5.3538	9.484E-03	-5.120E-04			1.08
All ages	Adenovirus	Mean	0.0374	-6.692E-01	9.606E-02	-5.3428	8.841E-03	-5.427E-03			1.05
All ages	Adenovirus	Prop pos	0.2156	-3.856E-01	9.364E-02	-5.3363	1.016E-02	-1.132E-02			1.16
All ages	Enterovirus	Maximum	0.2442	8.070E-03	5.000E-05	-5.3968	1.158E-02	-7.700E-05			1.23
All ages	Enterovirus	Mean	0.259	1.079E-01	8.850E-03	-5.3968	1.160E-02	-1.094E-03			1.21
All ages	Enterovirus	Prop pos	0.2524	4.318E-01	1.380E-01	-5.4224	1.306E-02	-1.249E-02			1.25
All ages	GI Norovirus	Maximum	0.0687	6.330E-03	1.000E-05	-5.4112	8.363E-03	-5.300E-05			1.34
All ages	GI Norovirus	Mean	0.0663	9.689E-02	2.620E-03	-5.4114	8.179E-03	-7.960E-04			1.41
All ages	GI Norovirus	Prop pos	0.0135	1.013E+00	1.527E-01	-5.4222	6.987E-03	-5.929E-03	0.2346	1.25182	1.44
Adults	All	Maximum	0.0084	1.234E-02	2.000E-05	-5.4494	1.512E-02	-1.370E-04	19.3238	1.27594	1.99
Adults	All	Mean	0.0101	1.715E-01	4.000E-03	-5.4539	1.593E-02	-2.094E-03	1.4285	1.2552	1.99
Adults	All	Prop pos	0.1143	5.745E-01	1.262E-01	-5.5043	3.215E-02	-2.982E-02	0.2521	1.34552	1.44
Adults	Adenovirus	Maximum	0.7278	-1.554E-02	1.960E-03	-5.3594	2.075E-02	-1.117E-03			1
Adults	Adenovirus	Mean	0.5887	-2.549E-01	2.185E-01	-5.3526	2.032E-02	-1.301E-02			1.02
Adults	Adenovirus	Prop pos	0.3772	-4.156E-01	2.162E-01	-5.3171	2.100E-02	-2.582E-02			1.13
Adults	Enterovirus	Maximum	0.0277	2.138E-02	9.000E-05	-5.4073	2.277E-02	-1.650E-04	13.2704	1.33629	1.84
Adults	Enterovirus	Mean	0.0296	2.920E-01	1.665E-02	-5.4080	2.294E-02	-2.361E-03	0.7627	1.29815	1.79
Adults	Enterovirus	Prop pos	0.0741	9.987E-01	2.953E-01	-5.4619	2.628E-02	-2.763E-02			1.78
Adults	GI Norovirus	Maximum	0.0427	1.047E-02	2.000E-05	-5.4151	1.443E-02	-1.210E-04	30.458	1.38084	1.55
Adults	GI Norovirus	Mean	0.0338	1.653E-01	5.620E-03	-5.4169	1.386E-02	-1.818E-03	2.0726	1.38592	1.67
Adults	GI Norovirus	Prop pos	0.0026	1.787E+00	3.054E-01	-5.4356	1.086E-02	-1.323E-02	0.2165	1.39596	1.88
Adults	Enterovirus Periods 3&4 only	Mean	0.0410	3.628E-01	2.513E-02	-5.5812	7.996E-03	-3.990E-03	1.01	1.55	1.84

Supplemental Table S3 (cont)

			Linear Fit [a]						Spline Fit [a]		
Participant Age Group	Virus Type	Virus Exposure Measure	P-value for Beta	Beta	Beta Variance	Intercept	Intercept Variance	Beta-Intercept Covariance	Threshold Point for Significant IRR	IRR at Significant Virus Threshold Point	Max IRR [b]
Children ≤ 12	All	Maximum	0.4548	2.290E-03	1.000E-05	-5.4150	6.963E-03	-5.600E-05			1.22
Children ≤ 12	All	Mean	0.6336	2.085E-02	1.880E-03	-5.4111	7.283E-03	-8.710E-04			1.18
Children ≤ 12	All	Prop pos	0.965	1.025E-02	5.370E-02	-5.4046	1.021E-02	-1.212E-02			1.18
Children ≤ 12	Adenovirus	Maximum	0.0192	-7.517E-02	9.400E-04	-5.3578	5.554E-03	-4.680E-04			1.14
Children ≤ 12	Adenovirus	Mean	0.0038	-9.039E-01	8.547E-02	-5.3446	5.177E-03	-4.537E-03			1.11
Children ≤ 12	Adenovirus	Prop pos	0.1994	-3.754E-01	8.255E-02	-5.3546	6.642E-03	-9.824E-03	0.1225	1.15189	1.18
Children ≤ 12	Enterovirus	Maximum	0.9941	5.000E-05	5.000E-05	-5.4025	7.302E-03	-7.900E-05			1
Children ≤ 12	Enterovirus	Mean	0.9045	-1.193E-02	9.750E-03	-5.4011	7.273E-03	-1.099E-03			1
Children ≤ 12	Enterovirus	Prop pos	0.9452	2.596E-02	1.407E-01	-5.4046	8.412E-03	-1.253E-02			1
Children ≤ 12	GI Norovirus	Maximum	0.2228	3.980E-03	1.000E-05	-5.4171	6.111E-03	-4.500E-05			1.2
Children ≤ 12	GI Norovirus	Mean	0.2316	5.973E-02	2.410E-03	-5.4171	6.037E-03	-7.010E-04			1.27
Children ≤ 12	GI Norovirus	Prop pos	0.0918	6.674E-01	1.487E-01	-5.4275	5.236E-03	-5.189E-03			
Children < 5	All	Mean									1.42
Children < 5	All	Prop pos	0.7175	1.222E-01	1.123E-01	-4.9421	1.181E-02	-2.509E-02			1.25
Children < 5	Adenovirus	Maximum	0.5074	-3.198E-02	2.280E-03	-4.8983	7.170E-03	-1.114E-03			1.3
Children < 5	Adenovirus	Mean	0.1129	-8.004E-01	2.430E-01	-4.8709	8.654E-03	-1.219E-02			1.3
Children < 5	Adenovirus	Prop pos	0.557	-2.445E-01	1.701E-01	-4.8870	9.144E-03	-1.954E-02	0.098	1.28703	1.39
Children < 5	Enterovirus	Maximum	0.6611	-5.430E-03	1.500E-04	-4.9064	6.115E-03	-2.180E-04			1.15
Children < 5	Enterovirus	Mean	0.7096	-6.490E-02	2.990E-02	-4.9075	6.207E-03	-3.180E-03			1.1
Children < 5	Enterovirus	Prop pos	0.9906	7.260E-03	3.740E-01	-4.9152	9.024E-03	-3.222E-02			1.01
Children < 5	GI Norovirus	Prop pos	0.0886	1.034E+00	3.498E-01	-4.9630	4.542E-03	-1.540E-02			1.35

[a] A row of missing information in the table, linear fit or spline fit, indicates model convergence problems. There were three subgroups (children < 5/all viruses/maximum concentration, children < 5/GI norovirus/mean concentration, children < 5/GI norovirus/maximum concentration) where the adjusted models for both the linear and spline fits experienced convergence problems. These subgroups are not included in the table.

[b] If only the maximum IRR is displayed, this indicates that no threshold point was identified.

[c] Adjusted models included Normally distributed random intercepts (with mean=0) for community and surveillance period.

Supplemental Material Table S4. Virus types, frequencies, and concentrations by qPCR and frequencies of culturable adenovirus and enterovirus by ICC-qPCR for the subset of tap water samples collected during short-term chlorination in the communities (n = 86).

Virus Type	Number qPCR Positive Samples (%)	Virus Concentration (genomic copies/L)			Number ICC-qPCR Positive Samples (%)[b]
		Mean	95th Percentile[a]	Maximum	
Adenovirus	10 (12)	0.04	0.2	1	3/10 (30)
Enterovirus	3 (3)	0.007	0	0.5	0/3 (0)
GI Norovirus	8 (9)	0.5	1	26	
GII Norovirus	0 (0)	0	0	0	
Hepatitis A virus	1 (1)	0.0002	0	0.01	
Rotavirus	0 (0)	0	0	0	
All-viruses	20 (23)[c]	0.6	1.4	26	

[a] The median and 75th percentile concentrations for all sample groups were zero therefore the 95th percentile is reported.
[b] ICC-qPCR was performed only on qPCR positive samples.
[c] This number is less than the sum of virus types because some samples were positive for two or more viruses.

Supplemental Material Table S5. Number of AGI episodes and person-time of follow-up by age group, surveillance period, and community.

Age group	Period	Community ID	AGI episodes	Person-days	Person-years	Incidence (episodes/person-year)
All ages	1	1	62	12167	33.31	1.86
		2	30	5496	15.05	1.99
		3	24	5758	15.76	1.52
		4	64	10457	28.63	2.24
		5	25	4482	12.27	2.04
		6	62	11205	30.68	2.02
		7	65	10560	28.91	2.25
		8	70	6589	18.04	3.88
		9	38	5933	16.24	2.34
		10	35	5519	15.11	2.32
		11	46	6392	17.50	2.63
		12	78	12453	34.09	2.29
		13	41	8772	24.02	1.71
		14	36	6798	18.61	1.93
	2	1	49	11398	31.21	1.57
		2	23	5162	14.13	1.63
		3	27	5057	13.85	1.95
		4	33	8464	23.17	1.42
		5	15	3520	9.64	1.56
		6	43	10546	28.87	1.49
		7	28	9316	25.51	1.10
		8	27	6103	16.71	1.62
		9	20	5499	15.06	1.33
		10	22	4915	13.46	1.63
		11	34	5210	14.26	2.38

Supplemental Material Table S5						
**Age group**	**Period**	**Community ID**	**AGI episodes**	**Person-days**	**Person-years**	**Incidence (episodes/person-year)**
		12	36	11370	31.13	1.16
		13	25	7517	20.58	1.21
		14	27	5359	14.67	1.84
	3	1	49	11670	31.95	1.53
		2	21	5154	14.11	1.49
		3	13	5309	14.54	0.89
		4	37	7971	21.82	1.70
		5	22	3435	9.40	2.34
		6	42	9980	27.32	1.54
		7	35	8677	23.76	1.47
		8	27	4596	12.58	2.15
		9	24	5298	14.51	1.65
		10	29	4579	12.54	2.31
		11	26	5086	13.92	1.87
		12	42	10582	28.97	1.45
		13	38	7264	19.89	1.91
		14	25	5284	14.47	1.73
	4	1	47	10771	29.49	1.59
		2	10	5000	13.69	0.73
		3	26	5186	14.20	1.83
		4	26	7595	20.79	1.25
		5	15	2942	8.05	1.86
		6	33	9115	24.96	1.32
		7	18	7598	20.80	0.87
		8	24	3803	10.41	2.31
		9	24	4900	13.42	1.79
		10	15	4720	12.92	1.16

Supplemental Material Table S5						
**Age group**	**Period**	**Community ID**	**AGI episodes**	**Person-days**	**Person-years**	**Incidence (episodes/person-year)**
		11	13	4117	11.27	1.15
		12	34	9450	25.87	1.31
		13	28	7080	19.38	1.44
		14	15	4878	13.36	1.12
Adults	1	1	23	4677	12.80	1.80
		2	9	1691	4.63	1.94
		3	10	2100	5.75	1.74
		4	29	4054	11.10	2.61
		5	11	1458	3.99	2.76
		6	25	4131	11.31	2.21
		7	25	3608	9.88	2.53
		8	36	2167	5.93	6.07
		9	17	2329	6.38	2.67
		10	14	1980	5.42	2.58
		11	19	2539	6.95	2.73
		12	35	4419	12.10	2.89
		13	14	3366	9.22	1.52
		14	13	2330	6.38	2.04
	2	1	18	4224	11.56	1.56
		2	5	1498	4.10	1.22
		3	11	1789	4.90	2.25
		4	13	3186	8.72	1.49
		5	5	1185	3.24	1.54
		6	15	3881	10.63	1.41
		7	7	3259	8.92	0.78
		8	10	1939	5.31	1.88
		9	9	2091	5.72	1.57

Supplemental Material Table S5						
**Age group**	**Period**	**Community ID**	**AGI episodes**	**Person-days**	**Person-years**	**Incidence (episodes/person-year)**
		10	10	1737	4.76	2.10
		11	16	2159	5.91	2.71
		12	14	4001	10.95	1.28
		13	5	2883	7.89	0.63
		14	11	1870	5.12	2.15
	3	1	20	4138	11.33	1.77
		2	4	1477	4.04	0.99
		3	4	1836	5.03	0.80
		4	9	3033	8.30	1.08
		5	5	1125	3.08	1.62
		6	17	3518	9.63	1.76
		7	12	3037	8.31	1.44
		8	9	1556	4.26	2.11
		9	7	1966	5.38	1.30
		10	10	1563	4.28	2.34
		11	11	1944	5.32	2.07
		12	15	3560	9.75	1.54
		13	13	2750	7.53	1.73
		14	6	1816	4.97	1.21
	4	1	17	3878	10.62	1.60
		2	1	1447	3.96	0.25
		3	8	1857	5.08	1.57
		4	7	2945	8.06	0.87
		5	5	971	2.66	1.88
		6	8	3161	8.65	0.92
		7	9	2646	7.24	1.24
		8	8	1329	3.64	2.20

Supplemental Material Table S5						
**Age group**	**Period**	**Community ID**	**AGI episodes**	**Person-days**	**Person-years**	**Incidence (episodes/person-year)**
		9	12	1763	4.83	2.49
		10	7	1604	4.39	1.59
		11	5	1594	4.36	1.15
		12	12	3235	8.86	1.35
		13	12	2602	7.12	1.68
		14	3	1668	4.57	0.66
Children <=12 years	1	1	39	7490	20.51	1.90
		2	21	3805	10.42	2.02
		3	14	3658	10.02	1.40
		4	35	6403	17.53	2.00
		5	14	3024	8.28	1.69
		6	37	7074	19.37	1.91
		7	40	6952	19.03	2.10
		8	34	4422	12.11	2.81
		9	21	3604	9.87	2.13
		10	21	3539	9.69	2.17
		11	27	3853	10.55	2.56
		12	43	8034	22.00	1.95
		13	27	5406	14.80	1.82
		14	23	4468	12.23	1.88
	2	1	31	7174	19.64	1.58
		2	18	3664	10.03	1.79
		3	16	3268	8.95	1.79
		4	20	5278	14.45	1.38
		5	10	2335	6.39	1.56
		6	28	6665	18.25	1.53
		7	21	6057	16.58	1.27

Supplemental Material Table S5						
**Age group**	**Period**	**Community ID**	**AGI episodes**	**Person-days**	**Person-years**	**Incidence (episodes/person-year)**
		8	17	4164	11.40	1.49
		9	11	3408	9.33	1.18
		10	12	3178	8.70	1.38
		11	18	3051	8.35	2.15
		12	22	7369	20.18	1.09
		13	20	4634	12.69	1.58
		14	16	3489	9.55	1.67
	3	1	29	7532	20.62	1.41
		2	17	3677	10.07	1.69
		3	9	3473	9.51	0.95
		4	28	4938	13.52	2.07
		5	17	2310	6.32	2.69
		6	25	6462	17.69	1.41
		7	23	5640	15.44	1.49
		8	18	3040	8.32	2.16
		9	17	3332	9.12	1.86
		10	19	3016	8.26	2.30
		11	15	3142	8.60	1.74
		12	27	7022	19.23	1.40
		13	25	4514	12.36	2.02
		14	19	3468	9.49	2.00
	4	1	30	6893	18.87	1.59
		2	9	3553	9.73	0.93
		3	18	3329	9.11	1.97
		4	19	4650	12.73	1.49
		5	10	1971	5.40	1.85
		6	25	5954	16.30	1.53

Supplemental Material Table S5						
**Age group**	**Period**	**Community ID**	**AGI episodes**	**Person-days**	**Person-years**	**Incidence (episodes/person-year)**
		7	9	4952	13.56	0.66
		8	16	2474	6.77	2.36
		9	12	3137	8.59	1.40
		10	8	3116	8.53	0.94
		11	8	2523	6.91	1.16
		12	22	6215	17.02	1.29
		13	16	4478	12.26	1.31
		14	12	3210	8.79	1.37
Children < 5 years	1	1	24	3402	9.31	2.58
		2	6	983	2.69	2.23
		3	9	1393	3.81	2.36
		4	20	2020	5.53	3.62
		5	10	1063	2.91	3.44
		6	21	3047	8.34	2.52
		7	13	1287	3.52	3.69
		8	18	1675	4.59	3.93
		9	16	1241	3.40	4.71
		10	8	879	2.41	3.32
		11	19	2458	6.73	2.82
		12	16	2174	5.95	2.69
		13	18	2608	7.14	2.52
		14	13	1671	4.57	2.84
	2	1	18	2801	7.67	2.35
		2	3	804	2.20	1.36
		3	7	1259	3.45	2.03
		4	10	1416	3.88	2.58
		5	5	704	1.93	2.59

Supplemental Material Table S5						
**Age group**	**Period**	**Community ID**	**AGI episodes**	**Person-days**	**Person-years**	**Incidence (episodes/person-year)**
		6	17	2441	6.68	2.54
		7	9	771	2.11	4.26
		8	11	1394	3.82	2.88
		9	2	1082	2.96	0.68
		10	3	704	1.93	1.56
		11	15	1794	4.91	3.05
		12	6	1713	4.69	1.28
		13	10	2273	6.22	1.61
		14	8	1282	3.51	2.28
	3	1	17	2629	7.20	2.36
		2	4	813	2.23	1.80
		3	3	1049	2.87	1.04
		4	13	1127	3.09	4.21
		5	15	734	2.01	7.46
		6	13	2516	6.89	1.89
		7	7	489	1.34	5.23
		8	5	1049	2.87	1.74
		9	9	1044	2.86	3.15
		10	9	632	1.73	5.20
		11	8	1788	4.90	1.63
		12	8	1553	4.25	1.88
		13	17	1873	5.13	3.32
		14	8	867	2.37	3.37
	4	1	18	1722	4.71	3.82
		2	2	805	2.20	0.91
		3	9	949	2.60	3.46
		4	7	882	2.41	2.90

Supplemental Material Table S5						
**Age group**	**Period**	**Community ID**	**AGI episodes**	**Person-days**	**Person-years**	**Incidence (episodes/person-year)**
		5	8	569	1.56	5.14
		6	12	1730	4.74	2.53
		7	3	396	1.08	2.77
		8	8	653	1.79	4.47
		9	5	758	2.08	2.41
		10	6	675	1.85	3.25
		11	3	1074	2.94	1.02
		12	5	1014	2.78	1.80
		13	8	1709	4.68	1.71
		14	4	675	1.85	2.16

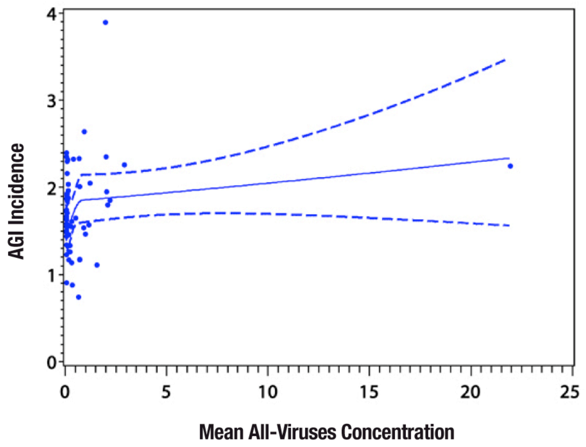

Supplemental Material Figure S2. Spline fit depicting the influence of an outlier on the association between AGI incidence, all ages, and all-viruses mean concentration. The outlier is a mean virus concentration value from one community that had unusually high NoV-GI concentrations during period 1. The model is unadjusted. The data are the same as Figure 2, panel A, in the manuscript except for inclusion of the outlier. Note the difference in the horizontal axis scales.

Supplemental Material Table S6. Virus types, frequencies, and concentrations by qPCR and frequencies of culturable adenovirus and enterovirus by ICC-qPCR for the well water samples collected immediately following UV disinfection before the water entered the distribution system (n = 191). These data represent the potential contribution of viruses from UV-treated well water to the tap water virus measurements.

Virus Type	Number qPCR Positive Samples (%)	Virus Concentration (genomic copies/L)			Number ICC-qPCR Positive Samples (%)[b]
		Mean	95th Percentile[a]	Maximum	
Adenovirus	17 (9)	0.02	0.1	1	3/17 (18)
Enterovirus	3 (2)	0.007	0	1	0/3 (0)
GI Norovirus	0 (0)	0	0	0	
GII Norovirus	0 (0)	0	0	0	
Hepatitis A virus	1 (0.5)	0.001	0	0.2	
Rotavirus	0 (0)	0	0	0	
All-viruses	19 (10)[c]	0.03	0.2	2	

[a] The median and 75th percentile concentrations for all sample groups were zero therefore the 95th percentile is reported.
[b] ICC-qPCR was performed only on qPCR positive samples.
[c] This number is less than the sum of virus types because some samples were positive for two or more viruses.

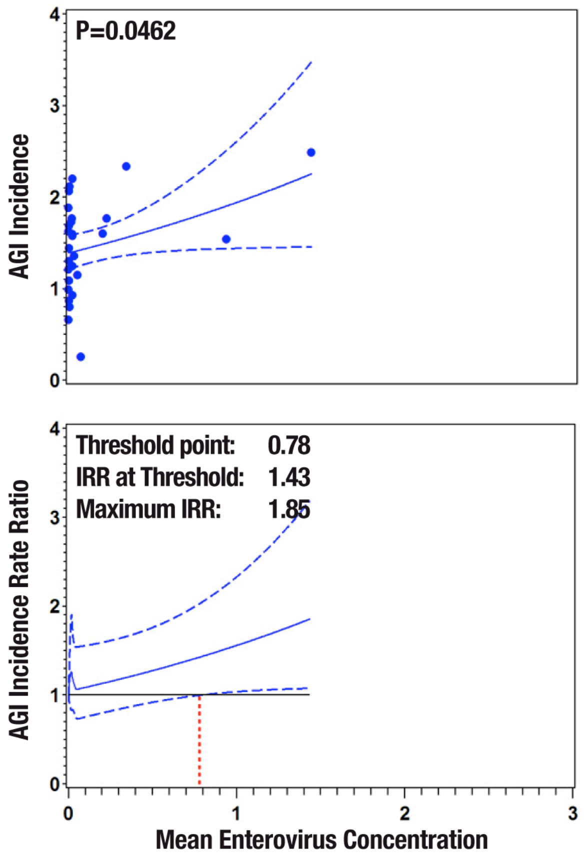

Supplemental Material Figure S3. Association between adult AGI incidence (episodes/person-year) and enterovirus mean concentration in tap water with the analysis restricted to surveillance periods 3 and 4 only; the models are unadjusted for community and period. Top plot: Linear (in the log of the AGI incidence) fit derived from Poisson regression. Each data point represents a community and period. Bottom plot: AGI incidence rate ratio (IRR, a measure of relative risk) based on a spline fit with the vertical red dashed line indicating the virus exposure threshold above which AGI risk was significantly elevated. Blue dashed lines in both top and bottom plots are the lower and upper 95% confidence limits. Enterovirus concentration reported as genomic copies/L. Regression coefficients are provided in Supplemental Material Table S3.

Statistical Models Sensitivity Analyses

We conducted two post hoc sensitivity analyses for the models highlighted in Figure 2 of the manuscript. To evaluate the potential confounding effect of UV disinfection at the municipal wells, we fit models with and without a dichotomous variable indicating whether UV disinfection was in place and compared corresponding incidence rate ratio (IRR) estimates for the virus exposure measure. It was decided *a priori* that a ten percent difference in IRR would constitute meaningful confounding. Since the exposure measure was a continuous variable, it was necessary to select a relevant difference in the virus measures to use in the IRR computations (e.g., compute the IRR for a 1-unit difference in arithmetic mean virus concentration). We selected the differences based on the observed range of values for each virus measure. For arithmetic mean virus concentration, a 1-unit difference was used. The corresponding values for proportion of virus-positive tap water samples and maximum virus concentration were 0.10 and 10 units respectively.

The results of the confounding analysis are reported in Supplemental Material Table S7. Among the nine models examined, the percent change in the IRR when UV status was included in the model ranged between -0.8% and 2.3%. We conclude the confounding effect of UV disinfection was minimal on the virus exposure –AGI incidence associations.

We also conducted analyses where outcome and exposure data were aggregated at the level of calendar month within community and surveillance period. The first surveillance period in 2006 spanned April through June and a portion of July. Since there was limited follow-up time in July (only the first two days), outcome and exposure data for June and July of 2006 were combined in the monthly analyses. Specifications for the Poisson regression models were the same as the primary analyses except that the general overdispersion component was replaced by

a component that accommodated the correlation of the monthly measurements within community and surveillance period.

The outcome and exposure data exhibited substantially more variability when aggregated at the level of the month within community and surveillance period as compared to the primary analyses where data were aggregated at the level of 12-week surveillance periods. This was manifested in higher p-values and dampened incidence rate ratios (Supplemental Material Table S8). In the monthly analyses, one of the subgroups highlighted in Figure 2 maintained statistical significance at the .05 level with the linear representation of the virus concentration effect (Panel D-proportion of tap water samples positive for GI norovirus) and three achieved statistically significant threshold points in the spline analyses (Panel C-mean GI norovirus concentration, Panel D-proportion of tap water samples positive for GI norovirus, and Panel E-maximum GI norovirus concentration). The proportion of tap water samples positive for GI norovirus among children aged <5 in surveillance period 1(Panel F) attained marginal significance in the monthly analyses with the linear representation (p=.11). In the spline analyses for this subgroup, the estimated incidence rate ratio was ≥ the null value of 1.0 throughout the range of virus concentration values, but did not achieve statistical significance at the .05 level. It is possible that the less stable monthly data are more accurately represented by splines. For reasons stated in the manuscript, we feel strongly that aggregation of the exposure data at the level of 12-week surveillance periods provides the most objective and accurate representation of virus levels in the communities.

Supplemental Material Table S7. Examination of potential confounding by UV disinfection status on the virus exposure –AGI incidence associations. Data aggregated as in the primary analyses, by community and surveillance period. Post hoc analyses restricted to those models shown in Figure 2 of the manuscript.

Participant Age Group	Virus Type	Virus Exposure Measure	Adjusted Model?	UV Status in Regression Model	P-value for Virus Exposure Effect	Incidence Rate Ratio for X-Unit Difference[a]	Percent Change in Incidence Rate Ratio
Adults	Enterovirus	Mean	No	No	0.0462	1.40124	.
Adults	Enterovirus	Mean	No	Yes	0.0422	1.40374	0.2
Adults	Enterovirus	Mean	Yes	No	0.0296	1.33905	.
Adults	Enterovirus	Mean	Yes	Yes	0.0338	1.33566	-0.3
All ages	Adenovirus	Mean	Yes	No	0.0374	0.51211	.
All ages	Adenovirus	Mean	Yes	Yes	0.0377	0.50791	-0.8
All ages	All	Mean	No	No	0.0093	1.13853	.
All ages	All	Mean	No	Yes	0.003	1.16425	2.3
All ages	All	Mean	Yes	No	0.0977	1.07825	.
All ages	All	Mean	Yes	Yes	0.0584	1.09722	1.8
All ages	GI Norovirus	Maximum	No	No	0.0011	1.11439	.
All ages	GI Norovirus	Maximum	No	Yes	0.0003	1.12996	1.4
All ages	GI Norovirus	Mean	No	No	0.0006	1.18807	.
All ages	GI Norovirus	Mean	No	Yes	0.0002	1.21411	2.2
All ages	GI Norovirus	Proportion positive	No	No	<.0001	1.19149	.
All ages	GI Norovirus	Proportion positive	No	Yes	<.0001	1.19652	0.4
Children < 5	GI Norovirus	Mean	No	No	0.005	1.17633	.
Children < 5	GI Norovirus	Mean	No	Yes	0.0161	1.17147	-0.4

[a] X = 1 for mean, X = 10 for maximum, X = 0.1 for proportion positive

Supplemental Material Table S8. Poisson regression results with AGI and virus data aggregated at the level of community and calendar month. Post hoc analyses restricted to those models shown in Figure 2 of the manuscript. UV disinfection status is not included in the models, like the primary analyses.

Participant Age Group	Virus Type	Virus Exposure Measure	Adjusted Model	Time Aggregation Level	P-value for Beta	Beta	Incidence Rate Ratio for X-Unit Difference[a]	Percent Difference in IRR (1 month vs Period	Beta Variance
Adults	Enterovirus	Mean	No	Month	0.4243	0.0923	1.09669	-21.7	0.01311
Adults	Enterovirus	Mean	No	Period-CID	0.0462	0.33736	1.40124		0.02596
Adults	Enterovirus	Mean	Yes	Month	0.2999	0.08067	1.08401	-19.0	0.00601
Adults	Enterovirus	Mean	Yes	Period-CID	0.0296	0.29196	1.33905		0.01665
All ages	Adenovirus	Mean	Yes	Month	0.7056	-0.08263	0.92069	79.8	0.04762
All ages	Adenovirus	Mean	Yes	Period-CID	0.0374	-0.66921	0.51211		0.09606
All ages	All	Mean	No	Month	0.4525	0.02479	1.02510	-10.0	0.00108
All ages	All	Mean	No	Period-CID	0.0093	0.12974	1.13853		0.00231
All ages	All	Mean	Yes	Month	0.9689	0.00122	1.00122	-7.1	0.00097
All ages	All	Mean	Yes	Period-CID	0.0977	0.07534	1.07825		0.00197
All ages	GI Norovirus	Maximum	No	Month	0.3226	0.00556	1.05717	-5.1	0.00003
All ages	GI Norovirus	Maximum	No	Period-CID	0.0011	0.01083	1.11438		0.00001
All ages	GI Norovirus	Mean	No	Month	0.1953	0.04977	1.05103	-11.5	0.00146
All ages	GI Norovirus	Mean	No	Period-CID	0.0006	0.17233	1.18807		0.00225
All ages	GI Norovirus	Proportion positive	No	Month	0.0025	0.9178	1.09612	-8.0	0.08723
All ages	GI Norovirus	Proportion positive	No	Period-CID	<.0001	1.75203	1.19149		0.12896
Children < 5	GI Norovirus	Mean	No	Month	0.1052	0.0887	1.09275	-7.1	0.00274
Children < 5	GI Norovirus	Mean	No	Period-CID	0.005	0.1624	1.17633		0.00216

[a] X = 1 for mean, X = 10 for maximum, X = 0.1 for proportion positive

Supplemental Material Table S8 continued

Participant Age Group	Virus Type	Virus Exposure Measure	Adjusted Model	Time Aggregation Level	Intercept	Intercept Variance	Beta-Intercept Covariance	Threshold Point for Significant IRR	IRR at Significant Virus Threshold Point	Maximum IRR in Observed Data Range
Adults	Enterovirus	Mean	No	Month	-5.58622	0.005156	-0.002190909	.	.	1.66
Adults	Enterovirus	Mean	No	Period-CID	-5.57365	0.004145	-0.004507193	0.7843	1.42698	1.85
Adults	Enterovirus	Mean	Yes	Month	-5.40583	0.019738	-0.00088031	.	.	1.54
Adults	Enterovirus	Mean	Yes	Period-CID	-5.40799	0.022941	-0.00236087	0.7627	1.29815	1.79
All ages	Adenovirus	Mean	Yes	Month	-5.39256	0.009414	-0.00275755	.	.	1.1
All ages	Adenovirus	Mean	Yes	Period-CID	-5.34282	0.008841	-0.005427078	.	.	1.05
All ages	All	Mean	No	Month	-5.40531	0.00188	-0.000511292	.	.	1.21
All ages	All	Mean	No	Period-CID	-5.43585	0.002005	-0.001194315	1.8742	1.22256	1.52
All ages	All	Mean	Yes	Month	-5.39845	0.009733	-0.000430175	.	.	1.09
All ages	All	Mean	Yes	Period-CID	-5.41953	0.00972	-0.000964597	2.0056	1.19839	1.46
All ages	GI Norovirus	Maximum	No	Month	-5.40421	0.001726	-0.000064993	44.8128	1.77724	1.84
All ages	GI Norovirus	Maximum	No	Period-CID	-5.42417	0.00157	-0.00005258	14.7229	1.32648	1.5
All ages	GI Norovirus	Mean	No	Month	-5.40723	0.001707	-0.000444473	1.2077	1.41087	1.73
All ages	GI Norovirus	Mean	No	Period-CID	-5.42705	0.001543	-0.00079759	0.9851	1.29488	1.63
All ages	GI Norovirus	Proportion positive	No	Month	-5.42474	0.00152	-0.003664622	0.432	1.43892	2.41
All ages	GI Norovirus	Proportion positive	No	Period-CID	-5.43989	0.001339	-0.005729144	0.126	1.22955	1.87
Children < 5	GI Norovirus	Mean	No	Month	-4.89502	0.00497	-0.00199623	.	.	1.78
Children < 5	GI Norovirus	Mean	No	Period-CID	-4.95755	0.003465	-0.001861433	1.2071	1.26783	1.51

References

Ishiko H, Shimada Y, Yonaha M, Hashimoto O, Hayashi A, Sakae K, Takeda N. 2002. Molecular diagnosis of human enteroviruses by phylogeny-based classification by use of the VP4 sequence. J Infect Dis 185:744-754.

Kaufman JS. 2008. Social Epidemiology. In: Modern Epidemiology, (Rothman KJ, Greenland S, Lash TL eds). Philadelphia: Lippincott, Williams & Wilkins, 532-548.

Lambertini E, Spencer SK, Bertz PD, Loge FJ, Kieke BA, Borchardt MA. 2008. Concentration of enteroviruses, adenoviruses, and noroviruses from drinking water by use of glass wool filters. Appl Environ Microbiol 74:2990-2996.

Roche Molecular Biochemicals. 2000. Absolute quantification with external standards. Tech Note No. LC 11/2000, Roche Diagnostics GmbH, Mannheim, Germany.

Savitz DA, Olshan AF. 1995. Multiple comparisons and related issues in the interpretation of epidemiologic data. Am J Epidemiol 142:904-908.
